# USP30 deubiquitylates mitochondrial Parkin substrates and restricts apoptotic cell death

**DOI:** 10.15252/embr.201439820

**Published:** 2015-03-04

**Authors:** Jin-Rui Liang, Aitor Martinez, Jon D Lane, Ugo Mayor, Michael J Clague, Sylvie Urbé

**Affiliations:** 1 Cellular and Molecular Physiology, Institute of Translational Medicine, University of LiverpoolLiverpool, UK; 2 CIC Biogune, Bizkaia Teknologi ParkeaDerio, Spain; 3 IKERBASQUE, Basque Foundation for ScienceBilbao, Spain; 4 School of Biochemistry, University of BristolBristol, UK

**Keywords:** apoptosis, mitophagy, Parkin, TOM20, USP30

## Abstract

Mitochondria play a pivotal role in the orchestration of cell death pathways. Here, we show that the control of ubiquitin dynamics at mitochondria contributes to the regulation of apoptotic cell death. The unique mitochondrial deubiquitylase, USP30, opposes Parkin-dependent ubiquitylation of TOM20, and its depletion enhances depolarization-induced cell death in Parkin-overexpressing cells. Importantly, USP30 also regulates BAX/BAK-dependent apoptosis, and its depletion sensitizes cancer cells to BH3-mimetics. These results provide the first evidence for a fundamental role of USP30 in determining the threshold for mitochondrial cell death and suggest USP30 as a potential target for combinatorial anti-cancer therapy.

## Introduction

Mitochondria play a central role in metabolism and energy production as well as in the orchestration of apoptotic cell death. The key event in this process is mitochondrial outer-membrane permeabilization (MOMP), caused by activation, insertion and oligomerization of the pro-apoptotic multi-BH-domain proteins, BAX and BAK. These are antagonized and sequestered by anti-apoptotic BCL2, BCL-XL, BCL-w and/or MCL1 1, 2. A third class of pro-apoptotic BH3-only proteins antagonize the anti-apoptotic BCL2 family members and promote oligomerization of BAX and BAK, triggering MOMP. It is the relative availability of the various components of this precarious interaction equilibrium that dictate the ultimate outcome of cellular life or death. MOMP results in the release of cytochrome C into the cytosol and subsequent assembly of the caspase 9 apoptosome. Activation of BAX and BAK can be triggered by different types of stress (e.g. DNA damage), or by activation of the extrinsic apoptotic pathway through engagement of cell death receptors in association with caspase 8. Both initiating caspases, 9 and 8, can cleave and activate the effector caspases 7, 6 and 3, which in turn cleave PARP to generate a diagnostically useful fragment.

Mitochondrial dysfunction has been linked to neurodegeneration, in particular in the context of Parkinson's disease (PD), which is characterized by the loss of dopaminergic neurons from the substantia nigra. The aetiology of PD is diverse*,* but the large list of PD-associated genes includes at least three, whose function is linked to mitochondria: Parkin (*PARK2*), PINK1 (*PARK6*) and DJ1 (*PARK7*) 3. PINK1 and Parkin are involved in the clearance of damaged mitochondria by mitophagy, which can be observed upon acute mitochondrial depolarization 4. PINK1 is constitutively cleaved by mitochondrial inner-membrane (MIM)-associated proteases and degraded by the proteasome. Loss of the membrane potential leads to its accumulation on the mitochondrial outer membrane where it promotes recruitment and activation of Parkin by phosphorylating the Parkin UBL-domain as well as ubiquitin itself on a positionally equivalent serine 5, 6, 7. Both these phosphorylation events are essential for activation of Parkin and its ability to ubiquitylate its mitochondrial outer-membrane (MOM) substrates, which include MIRO, mitofusin (MFN), and the translocases TOM20 and 22. Ubiquitylated MOM proteins can act as receptors for the LC3-adapter p62/SQSTM1, promoting safe engulfment and degradation of damaged mitochondria in autolysosomes. Some MOM proteins, including MIRO, MFN and TOM20, can also be extracted from the membrane and subsequently targeted for degradation by the proteasome 8, 9, 10.

In most cells, Parkin levels are limiting and the signature bulk clearance of mitochondria is observed upon over-expressing high levels of heterologous Parkin. Here, we show that high levels of Parkin sensitize hTERT-RPE1 cells to CCCP-induced, PINK1-dependent cell death. We demonstrate that the unique MOM deubiquitylase (DUB), USP30, directly counteracts this manifestation of Parkin activity. Importantly, our data suggest that the link between USP30 and cell death pathways extends beyond this setting, as its depletion also sensitizes cancer cells against cell death induced by BH3-mimetics.

## Results and Discussion

### High expression levels of Parkin sensitize cells to depolarization-induced apoptotic cell death

**Figure 1:**
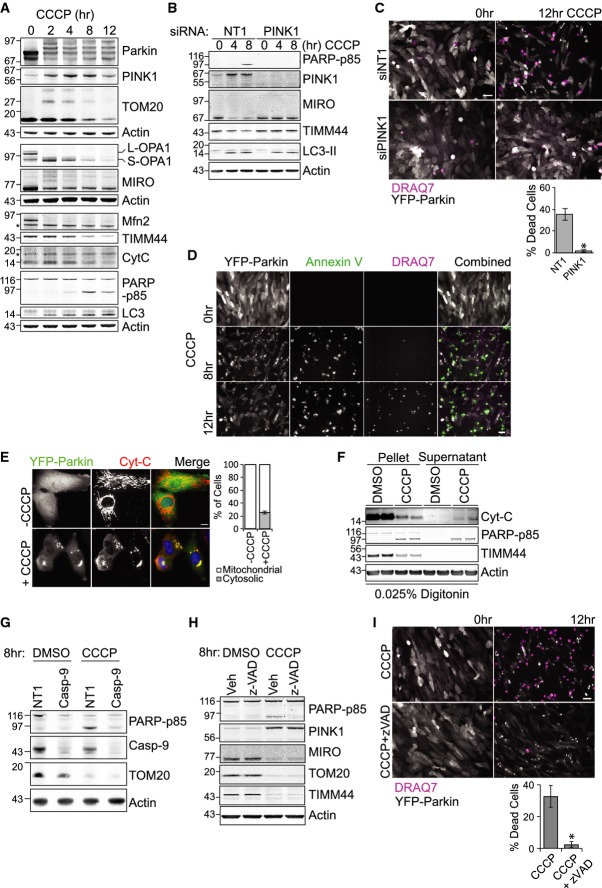
Parkin overexpression induces CCCP-dependent mitophagy and apoptotic cell death A hTERT-RPE1-YFP-Parkin cells treated with CCCP (10 μM), harvested and probed as indicated. * indicates non-specific bands.

YFP-Parkin-overexpressing hTERT-RPE1 cells undergo rapid and near-complete mitophagy in response to CCCP treatment, such that 80–90% of cells lose all of their mitochondria over a 24-h time course 11. We monitor this process using a panel of marker proteins, which report on distinct stages of mitophagy: initiation of mitophagy by depolarization is accompanied by cleavage of the inner-membrane protein L-OPA1 to S-OPA1, and accumulation of PINK1 and of higher molecular weight, ubiquitylated species of (YFP-)Parkin (Fig1A and Supplementary Fig S1A and B). This promotes degradation of MFN, ubiquitylation and subsequent degradation of MIRO and a gradual decrease of MOM (TOM20, TOM22) as well as MIM translocase subunits (TIMM44). TIMM44 serves as an unambiguous indicator for complete mitophagy, as it cannot be extracted for proteasomal degradation in contrast to MOM markers. Despite a strong accumulation of PINK1 in response to CCCP treatment, both MIRO and TIMM44 remain stable in parental RPE1 cells that do not overexpress Parkin (Supplementary Fig S1B).

B, C hTERT-RPE1-YFP-Parkin cells transfected with PINK1 or a non-targeting siRNA (NT1) for 72 h were treated with CCCP. (C) The progression of mitophagy was recorded by live-cell imaging in the presence of the membrane-impermeable dye DRAQ7 (pink). Single frames from a representative video (Supplementary Movie S1), YFP-Parkin (grey). Scale bar: 40 μm. Graph shows averaged data (% of DRAQ7-positive cells) from three independent experiments ± SD, two-tailed paired *t*-test, *P = 0.05.

D hTERT-RPE1-YFP-Parkin cells were treated with CCCP and imaged at 15-min intervals in the presence of the membrane-impermeable dye DRAQ7 (pink) and Alexa Fluor 350-conjugated annexin V (green). Single frames from a representative video, YFP-Parkin (grey). Scale bar: 40 μm.

E hTERT-RPE1-YFP-Parkin cells were treated with CCCP for 6 h, fixed and stained for Cyt-C. Cell with partially cytosolic Cyt-C staining is outlined. Scale bar: 10 μm. Graph shows percentage of cells with exclusively mitochondrial Cyt-C or partially cytosolic Cyt-C staining from three independent experiments ± SD.

F hTERT-RPE1-YFP-Parkin cells were treated with CCCP for 6 h and then harvested for digitonin fractionation. Duplicate samples of pellet and supernatant fractions were loaded at 1:3 (V:V).

G hTERT-RPE1-YFP-Parkin cells were transfected with NT1 or caspase 9 siRNA for 72 h and treated with CCCP for 8 h.

H hTERT-RPE1-YFP-Parkin cells were treated with CCCP and z-VAD-FMK (20 μM) for 8 h.

I hTERT-RPE1-YFP-Parkin cells treated as in (G) were imaged every 15 min in the presence of DRAQ7 (pink). Single frames from a representative video, YFP-Parkin (grey). Scale bar: 40 μm. Graph shows averaged data (% of DRAQ7-positive cells) from three independent experiments ± SD. Two-tailed paired *t*-test, *P = 0.05. Source data are available online for this figure.

We consistently observed a significant amount of apoptotic cell death in YFP-Parkin-overexpressing RPE1 cells, characterized by the appearance of cleaved PARP that was most apparent after 8 h of CCCP treatment (Fig1A). As an alternative method of mitochondrial depolarization, we used a combination of oligomycin A and antimycin A, inhibitors of the mitochondrial respiratory chain (Supplementary Fig S1A), which similarly induced MIRO, TOM22 and TIMM44 degradation as well as PARP cleavage. Depletion of PINK1 completely abolished MIRO as well as TIMM44 degradation and importantly, rescued cells from depolarization-induced apoptosis (Fig1B). This was also apparent in live-cell imaging experiments in which we monitor cell death with the cell-impermeable dye DRAQ7 (Fig1C and Supplementary Movie S1) and the apoptotic marker annexin V (Fig1D). Apoptotic cell death induced by intrinsic signals is usually accompanied by cytochrome C release from mitochondria and subsequent activation of caspase 9. Indeed, cytochrome C staining became partially cytosolic 6 h after depolarization in 25% of cells and leakage of cytochrome C into the cytosol was also apparent in the supernatant of digitonin-permeabilized cells after centrifugation (Fig1E and F). As predicted, siRNA-mediated depletion of caspase 9 prevented CCCP-induced cell death but did not inhibit TOM20 degradation (Fig1G). The pan-caspase inhibitor z-VAD also blocks CCCP-induced cell death, without interfering with degradation of MIRO, TOM20 or TIMM44 (Fig1H and I). Thus, Parkin-induced cell death is not a prerequisite for mitophagy in these cells.

Current data suggest a predominantly cytoprotective role for both Parkin and PINK1: loss-of-function mutations of Parkin and PINK1 have been associated with the loss of dopaminergic neurons in the substantia nigra in PD patients 3. Conversely, overexpression of Parkin in *Drosophila* neurons has been proposed to increase longevity 12. Our results suggest that high levels of Parkin expression in epithelial cells can promote apoptotic cell death, calling for caution with strategies to elevate Parkin expression levels as a therapeutically viable approach to treat PD. Interestingly a recent report suggests that PINK1 and Parkin can promote an apoptotic response to irreversible mitochondrial damage caused by valinomycin, through ubiquitin-dependent downregulation of MCL1 levels 13. In our system, CCCP-induced activation of Parkin did not lead to a decrease, but rather an accumulation of MCL1 (see further below, Fig 4C).

### Proteasome inhibitors protect against Parkin and PINK1-dependent cell death

Yoshii and colleagues reported that overexpression of Parkin in mouse embryonic fibroblasts results in mitochondrial membrane rupture that can be prevented with proteasome inhibitors 10. Whilst the authors did not report apoptosis in their system, they proposed that rapidly ubiquitylated MOM proteins, including MIRO, MFN and TOM20, are extracted by the AAA-ATPase p97/VCP and then degraded by the proteasome, causing a breach in the outer mitochondrial membrane. We wondered whether such ensuing damage could be the cause of the cell death we observe.

**Figure 2:**
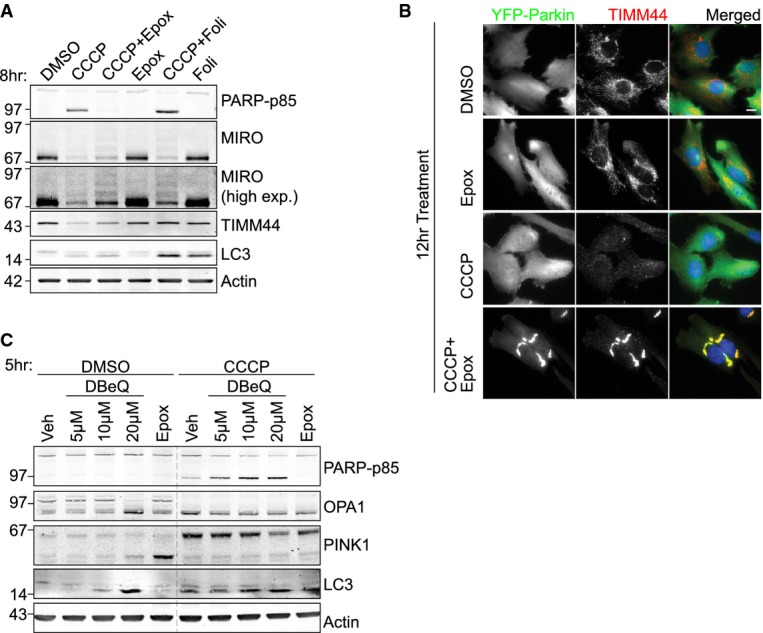
Proteasome but not lysosomal inhibitors prevent CCCP-induced cell death hTERT-RPE1-YFP-Parkin cells were treated with either CCCP, epoxomicin (Epox, 100 nM), folimycin (Foli, 100 nM) or a combination for 8 h. High exp: high exposure.

Simultaneous treatment of cells with CCCP and the proteasome inhibitor, epoxomicin, rescued a significant proportion of MIRO, some of which accumulated as a higher molecular weight ubiquitylated ladder (Fig2A). Both the pattern and degree of rescue were comparable with the vacuolar ATPase inhibitor, folimycin, suggesting that both proteasomal and autophagosomal/lysosomal activities contribute to MIRO degradation. The MIM protein TIMM44 was, as expected, fully stabilized by folimycin, but surprisingly also partially recovered by epoxomicin treatment. Immunofluorescence microscopy confirmed that Parkin and TIMM44-positive perinuclear aggregates persist in these cells for up to 12 h after treatment (Fig2B). These aggregates also stained positive for p62, the ubiquitin-binding adapter, which allows recruitment of the autophagic machinery via interaction with LC3 (Supplementary Fig S2A). In control cells, LC3 is seen to fully engulf small fragments of Parkin-tagged mitochondria after 8 h of CCCP treatment, whereas in epoxomicin-treated cells, the mitochondria were retained in tight and stable perinuclear aggregates, which were incompletely decorated with LC3 (Supplementary Fig S2B). Some larger aggregates were also transiently apparent in control cells, but these were gradually consumed such that the majority of cells were devoid of mitochondria by the 12-h time point. This failure to degrade Parkin and p62-decorated mitochondria, together with the lack of TIMM44 degradation, suggests that proteasome activity may also be required for late stages of mitophagy. Importantly, inhibition of the proteasome, but not the lysosomal degradation pathway, completely abolished apoptotic cell death (Fig2A). This protection was temporary, and at later time points (> 18 h), the simultaneous treatment of CCCP and proteasome inhibitors caused extensive cell death (Supplementary Movie S2). In contrast to proteasome inhibition, an inhibitor of p97/VCP, DBeQ 14, enhanced cell death caused by CCCP treatment (Fig2C). This suggests that p97-mediated extraction of MOM proteins is unlikely the exclusive cause for PINK1-Parkin-mediated cell death.

hTERT-RPE1-YFP-Parkin cells were treated for 12 h with CCCP (10 μM), with or without epoxomicin (100 nM). Cells were fixed and stained with anti-TIMM44 followed by anti-rabbit AF594 (red) and DAPI (blue). Scale bar: 10 μm.

hTERT-RPE1-YFP-Parkin cells were treated with CCCP (10 μM), epoxomicin (100 nM) and DBeQ for 5 h. Source data are available online for this figure.

### USP30 counteracts Parkin by deubiquitylating MOM proteins

We thought it conceivable that ubiquitin-dependent mitophagy may be a reversible process under the control of select DUBs. Indeed, whilst this manuscript was in preparation, multiple DUBs have been shown to modulate Parkin-dependent mitophagy 15, 16, 17. USP30 is the most attractive candidate for this role: it is the only known DUB that encodes a mitochondrial targeting sequence, it is conserved in zebrafish, flies and worms 18, and it was the only DUB constitutively associated with mitochondrial membranes in a systematic localization survey of 66 GFP-tagged DUBs 19. The *Drosophila* orthologue had previously been shown to interact with MIRO, one of the recently validated substrates of Parkin 20, 21, 22. Depletion of USP30 with two independent siRNA oligos in YFP-Parkin-expressing RPE1 cells reproducibly caused a small but significant decrease in MIRO protein levels, without a consistent effect on its mRNA, suggesting that USP30 may affect MIRO post-translationally (Supplementary Fig S3A–C). Isoform-specific siRNA-mediated depletion showed that hTERT-RPE1 cells predominantly express MIRO2 (Supplementary Fig S3D).

**Figure 3:**
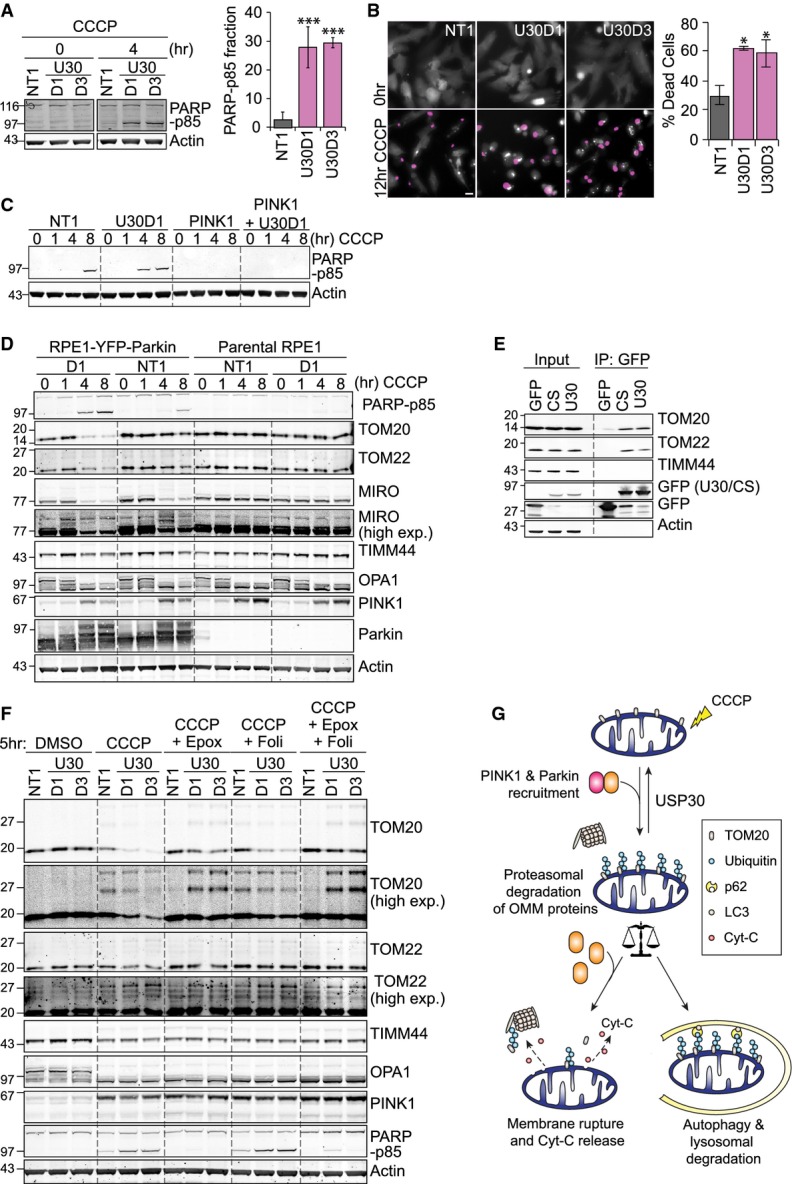
USP30 depletion enhances CCCP-induced Parkin-dependent cell death hTERT-RPE1-YFP-Parkin cells were transfected with non-targeting (NT1) or two individual USP30-targeting siRNA oligos (D1, D3) for 72 h and treated with CCCP for 4 h. PARP-p85 (normalized to actin) is shown as a mean ± SD of four independent experiments. One-way ANOVA, Dunnett's test, ***P = 0.001.

USP30 depletion has previously been found to favour an elongated, interconnected mitochondrial network 23; however, the most discernible and consistent phenotype of USP30 depletion in our cells was the enhancement of CCCP-induced cell death: PARP cleavage was clearly apparent after only 4 h of CCCP treatment of USP30-depleted cells, and dead cells accumulated faster in our live-cell imaging experiments (Fig3A and B and Supplementary Movie S3). The pro-apoptotic effect of USP30 depletion is strictly dependent on PINK1 and and overexpression of YFP-Parkin (Fig3C and D). In contrast, direct siRNA-mediated depletion of MIRO2 to comparable residual levels marginally decreased PARP cleavage, whilst a combined knockdown of USP30 and MIRO2 promoted cell death in a similar fashion to USP30 siRNA on its own (Supplementary Fig S3E). This suggests that the proapoptotic effect of USP30 depletion is not directly caused by enhanced MIRO destabilization in these cells.

hTERT-RPE1-YFP-Parkin cells were transfected and treated as in (A), and cells were imaged every 15 min in the presence of the membrane-impermeable dye DRAQ7 (pink). Shown is a cropped single frame from a representative video. YFP-Parkin shown in grey. Scale bar: 10 μm. Graph shows averaged data from three independent experiments ± SD. One-way ANOVA, Dunnett's test, *P = 0.05.

hTERT-RPE1-YFP-Parkin cells were transfected with siRNA against PINK1 and USP30 (D1) and treated with CCCP. Shown are results from a representative experiment.

hTERT-RPE1-YFP-Parkin cells and hTERT-RPE1 parental cells were transfected with NT1 or USP30 siRNA D1 and treated with CCCP. Shown are results from a representative experiment.

HEK293T cells expressing GFP-tagged USP30 were lysed and subjected to immunoprecipitation (IP) with anti-GFP and probed as indicated. 1.8% of input sample loaded. U30, USP30-GFP; CS, catalytically inactive mutant USP30-C77S-GFP.

hTERT-RPE1-YFP-Parkin cells were transfected with NT1 or USP30 siRNA (D1, D3) and then treated for 5 h with CCCP together with either epoxomicin (100 nM) or folimycin (100 nM).

Schematic diagram illustrating the role of the proteasome and USP30 in opposing mitophagy and CCCP-induced cell death in Parkin-overexpressing RPE1 cells. Source data are available online for this figure.

Amongst the mitochondrial proteins we analysed, only TOM20 and TOM22 showed strongly enhanced CCCP-induced degradation in USP30-depleted cells and both associate with USP30-GFP as well as its catalytically inactive mutant in a pull-down assay (Fig3D and E and Supplementary Fig S3F). Both proteins can be stabilized by proteasome inhibition, which allows for visualization of a specific enzyme–substrate relationship between USP30 and TOM20: (1) USP30 depletion increases the pool of di-ubiquitylated TOM20, which is rapidly degraded by the proteasome (Supplementary Fig S3E and F), and (2) mono- and di-ubiquitylated species of TOM20 can only be stabilized in USP30-depleted cells, suggesting that Parkin-dependent ubiquitylation of TOM20 is opposed by USP30 (Fig3F). TIMM44 or S-OPA1 degradation kinetics were not differentially affected, suggesting that USP30 depletion does not enhance the later stages of mitophagy.

Bingol and colleagues recently reported a role for overexpressed USP30 in counteracting Parkin-dependent mitophagy 15. In agreement with our results, their study identified TOM20 as a substrate of USP30. Most strikingly, they reported that USP30 depletion rescued flies suffering from Paraquat-induced symptoms that mimic neurodegenerative disorders (loss of dopamine release and motor dysfunction) and restored mitochondrial integrity and motor function in PINK1 and Parkin-deficient flies. In contrast, a systematic analysis of DUB knockdown phenotypes in *Drosophila* highlighted a protective role for USP30 during development: even incomplete suppression of USP30 expression caused developmental lethality or early death in adulthood 24.

Our data suggest that there is a fine balance between the proteasomal degradation of ubiquitylated MOM proteins and their engagement of the mitophagy machinery, which ensures the safe disposal of defective mitochondria (Fig3G). Proteasome inhibitors may delay CCCP-induced cell death caused by excessive Parkin activation by preventing mitochondrial membrane disintegration and cytochrome C release, whereas USP30 depletion enhances mitophagic clearance of damaged mitochondria but also has the potential to promote Parkin-induced cell death.

### USP30 depletion sensitizes cells to BH3-mimetics

**Figure 4:**
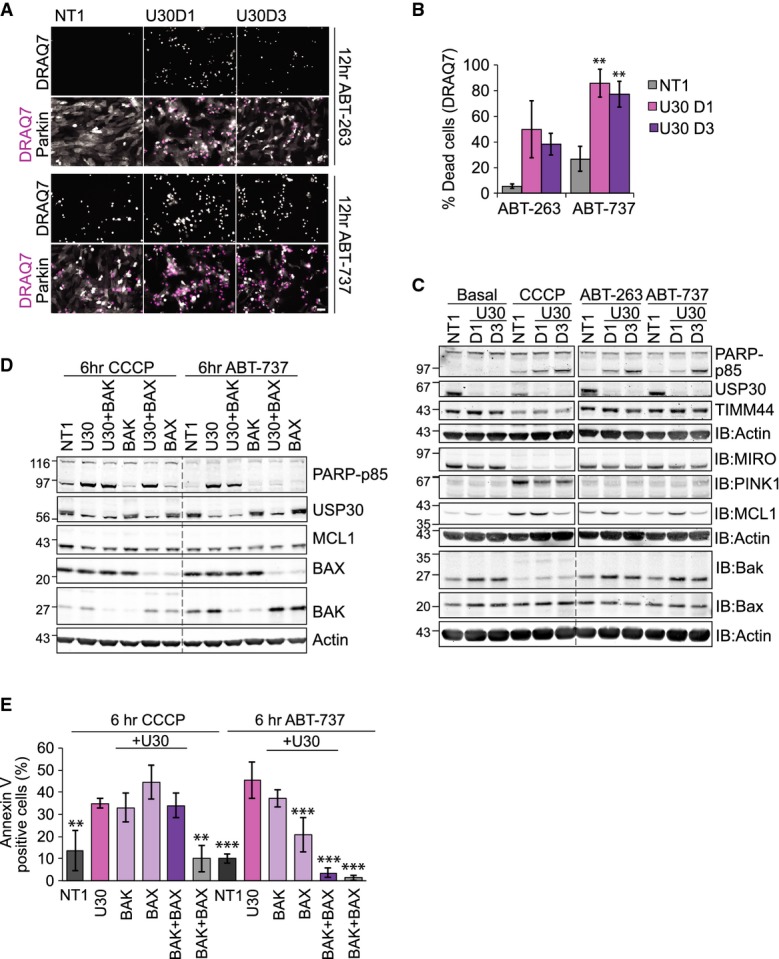
USP30 depletion sensitizes cells to BH3-mimetics hTERT-RPE1-YFP-Parkin cells were transfected for 72 h with NT1 or USP30 siRNA (D1 and D3) and then treated either with CCCP, ABT-263 or ABT-737 (all 10 μM). Cells were imaged at 30-min intervals in the presence of DRAQ7, and a single frame corresponding to the 12-h time point is shown (see also Supplementary Movies S3, S4 and S5). Top rows show the isolated DRAQ7 staining (dead cells in white on black). Bottom rows show YFP-Parkin in grey and DRAQ7 in pink. Scale bar: 40 μm.

Given the central position of mitochondria in stress-induced apoptosis, we wondered whether USP30 influenced other apoptotic scenarios. Synthetic BH3-mimetics, for example ABT-737 and ABT-263, promote apoptosis by mopping up free anti-apoptotic BCL2-family members, with the exception of MCL1 and BCL2A1, allowing BAX to form oligomers and trigger MOMP 25, 26, 27. These compounds have been shown to kill tumour cells. ABT-263 (Navitoclax) has shown early promise in the clinic; however, its success has largely been limited to cells expressing high BCL2 and low MCL1 protein levels 28. Treatment with ABT-737 and ABT-263 caused cell death in YFP-Parkin-expressing RPE1 cells within 8–12 h as assessed by the appearance of cells that incorporate DRAQ7 (Fig4A and B). Interestingly, USP30-depleted cells were sensitized not only to CCCP but also to ABT-263 and ABT-737-induced cell death (Fig4A–C, Supplementary Fig S4A–C and Supplementary Movies S4, S5 and S6). However, in this case, proteasome inhibition did not delay but rather further enhanced cell death (Supplementary Movies S4 and S5). Analysis of BCL2-family members showed that BAK protein, but not mRNA, levels were increased upon USP30 depletion with two individual siRNAs, whereas MCL1 expression levels were not consistently affected (Fig4C and D and Supplementary Fig S4D and E).

Graph shows the % of DRAQ7-positive cells after 12 h of treatment. ABT-263, 10 μM: *n *= 2, bars indicate range; ABT-737: *n *= 3 independent experiments, ± SD. One-way ANOVA and Dunnett's multiple comparison's test, **P = 0.01.

Same transfection and drug treatment as in (A). Cells were harvested after 6 h of CCCP or 4 h of ABT-263 and ABT-737 treatment and samples probed as indicated.

hTERT-RPE1-YFP-Parkin cells were transfected with NT1, or siRNAs targeting USP30 (D3), BAX and BAK for 72 h. Cells were treated with CCCP or ABT-737 for 6 h. Shown are samples from a representative experiment.

hTERT-RPE1-YFP-Parkin cells were transfected with siRNAs as indicated and treated with CCCP or ABT-737. Cells were imaged at hourly intervals in the presence of Alexa Fluor 350-conjugated annexin V. Graph shows the % of annexin V-positive cells at the 6-h time point, *n *= 3 independent experiments, ± SD. One-way ANOVA, Dunnett's test, comparison of all conditions versus USP30 knockdown, **P = 0.01, ***P = 0.001. Source data are available online for this figure.

**Figure 5:**
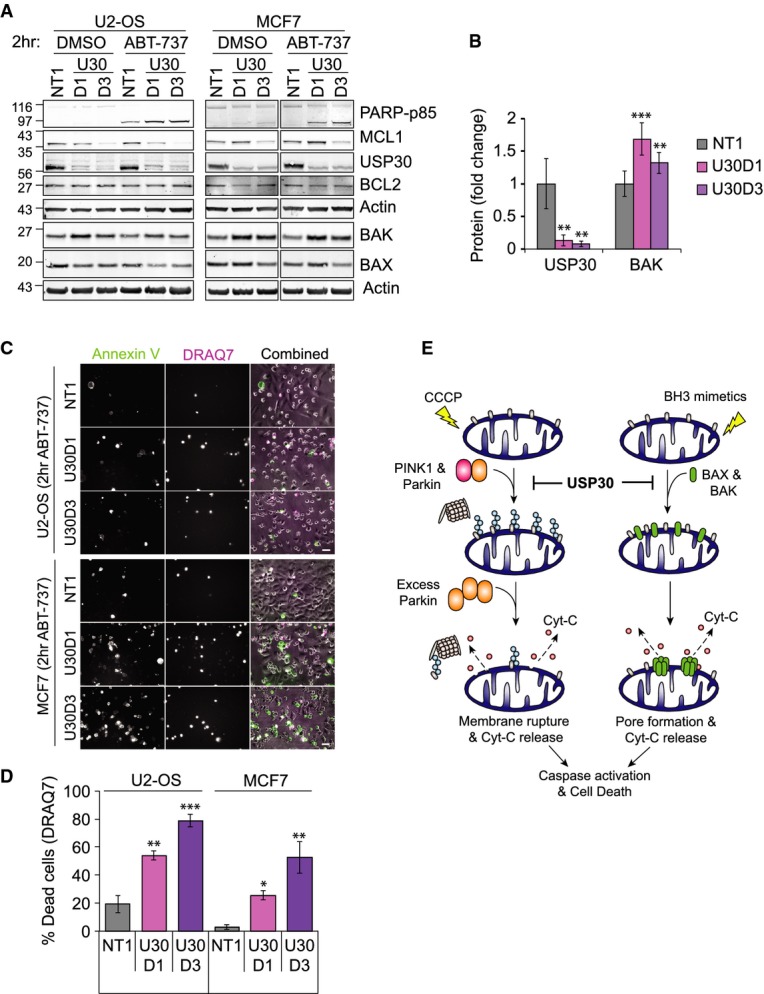
USP30 depletion sensitizes U2-OS and MCF7 cells to BH3-mimetics U2-OS and MCF7 cells were transfected for 72 h with NT1 or USP30 siRNA (D1 and D3), then incubated for 2 h with ABT-737. Shown are samples from a representative experiment.

Cell death promoted by USP30 depletion upon treatment with ABT-737 was partially suppressed by BAX knockdown but required concomitant depletion of both BAK and BAX for full suppression (Fig4D and E). In contrast, CCCP-induced apoptosis was insensitive to individual or simultaneous depletion of both BAK and BAX, consistent with a non-specific rupture of the mitochondrial membrane, triggered by excessive ubiquitylation and proteasomal degradation of outer-membrane proteins prior to the safe disposal of damaged mitochondria in autophagosomes. Interestingly, depletion of USP8, a DUB that has recently been proposed as a positive regulator of Parkin-mediated mitophagy, also promoted CCCP-induced and PINK1-dependent PARP cleavage, whilst mildly delaying TIMM44 degradation (Supplementary Fig S5A–D) 17. Neither USP15 (also recently suggested as a negative regulator of mitophagy) nor USP33 or AMSH (implicated in autophagic and endo-lysosomal processes, respectively) enhanced CCCP-induced cell death in Parkin-overexpressing RPE1 cells 16, 29, 30. Most strikingly, USP30 was unique amongst this DUB panel in sensitizing cells to ABT-737-induced apoptosis. This sensitization to mitochondrial apoptosis triggered by BH3-mimetics does not depend on Parkin overexpression but is also readily observed in U2-OS as well as in MCF7 cells (Fig5A–D, Supplementary Fig S4C, Supplementary Movies S7 and S8).

Graph shows BAK protein levels normalized to actin in U2-OS transfected as in (A), averaged data from three independent experiments, ± SD. One-way ANOVA and Dunnett's multiple comparison's test, **P = 0.01, ***P = 0.001.

U2-OS and MCF7 cells were transfected and treated as in (A) and then imaged in the presence of Alexa Fluor 350-conjugated annexin V (green) and DRAQ7 (pink). Shown are single frames (*t* = 2 h) of a representative video (Supplementary Movies S7 and S8). Scale bar: 40 μm.

Graph shows the percentage of DRAQ7-positive dead cells at *t* = 6 h for cells transfected and treated as in (C) (*n *= 3 independent experiments; ± SD; one-way ANOVA with Dunnett's test, comparison with NT1, *P = 0.05, **P = 0.01, ***P = 0.001).

Schematic diagram illustrating the dual role of USP30 in opposing cell death induced by an excess of Parkin-dependent ubiquitylation of MOM proteins and by BH3-mimetics. Source data are available online for this figure.

Our observations may suggest that USP30 deubiquitylates a central anti-apoptotic regulator to raise the threshold for apoptotic activation. Another DUB, USP9X, has previously been shown to deubiquitylate and stabilize MCL1, thereby reducing the sensitivity of cells to ABT-737 31. Our current data do not support a straightforward, direct role for USP30 in controlling MCL1 levels. We consistently observe an increase in BAK protein (Figs4C and D and 5A and B, Supplementary Fig S4E). This may, through direct association, contribute to reducing the active pool of MCL1 and thereby promote apoptotic death. However, this is unlikely to be the sole factor responsible for enhanced cell death since simultaneous depletion of BAK and BAX is required to fully suppress this effect. It is also conceivable that depletion of USP30 causes a low level of mitochondrial stress via an as-yet uncharacterized mechanism, rather than directly regulating the apoptotic machinery.

We have previously identified USP30 as one of the strongest hits in a siRNA-screen aimed at identifying DUBs involved in the HGF-induced cell scattering response in lung adenocarcinoma cells 32. This assay recapitulates some aspects of metastatic behaviour of cancer cells, and we proposed that essential regulators of this process, including USP30, might provide interesting new drug targets for anti-cancer therapies. Our new results, which indicate an anti-apoptotic role for USP30, only heighten this notion and suggest that USP30 may be a valuable target not just for the treatment of Parkinson's disease but also for combinatorial anti-cancer therapies.

## Materials and Methods

### Cell culture, transfection and RNA interference

Parental hTERT-RPE1, hTERT-RPE1-YFP-Parkin 11, SH-SY5Y, HEK293T and U2-OS cells were cultured in Dulbecco's modified Eagle's medium (DMEM)/F12 or DMEM (the latter 2) with 10% FBS, 1% non-essential amino acids and 1% penicillin/streptomycin. Reverse transfection with 40 nM siRNA using Lipofectamine RNAiMAX (Invitrogen) was carried out for 72 h. HEK293T cells were transfected for 24 h with pEGFP-C-GW, pEGFP-C3-USP30-siR1 and pEGFP-C3-USP30-C77S-siR1 using Genejuice (Novagen). Cell lysis and immunoprecipitation are described in the Supplementary Methods.

### Drug treatments

CCCP (10 μM; Sigma), oligomycin A and antimycin A (both 1 μM; Sigma), epoxomicin and folimycin (both 100 nM; Calbiochem), DBeQ (5–20 μM; Sigma), z-VAD-FMK (20 μM; Enzo Life Sciences), ABT-263 and ABT-737 (10 μM; Selleckchem).

### Immunofluorescence and live-cell microscopy

Cells were fixed using 4% paraformaldehyde in PBS, permeabilized with 0.2% Triton X-100 in PBS, prior to staining with AlexaFluor594 or 488-coupled antibodies and imaged using a Nikon Ti-Eclipse. Live cells were imaged using a Nikon Ti-Eclipse in an incubation chamber with 5% CO_2_ at 37°C. DRAQ7 (0.3 μM, CST) and AlexaFluor®350-conjugated annexin V (5 μl/ml; Invitrogen) were added to the medium. Images were captured at 15- or 30-min intervals using a CFI S Plan Fluor ELWD 20× objective and saved for playback in ImageJ at 3 frames/s.

### Statistics

*P*-values are indicated as *P = 0.05, **P = 0.01 and ***P = 0.001 and derived either by two-tailed paired *t*-test or, for multiple comparison analysis, by one-way ANOVA and Dunnett's *post hoc* test using GraphPad Prism 6.

For further methods, see Supplementary Methods.

## Supporting Information

Supplementary Legends

Review Process File
