# Supplementary figures and images for: USP30 deubiquitylates mitochondrial Parkin substrates and restricts apoptotic cell death
Source: EMBO Rep. 2015 Mar 4;16(5):618–27. doi: 10.15252/embr.201439820 (PMC4428036; doi:10.15252/embr.201439820)

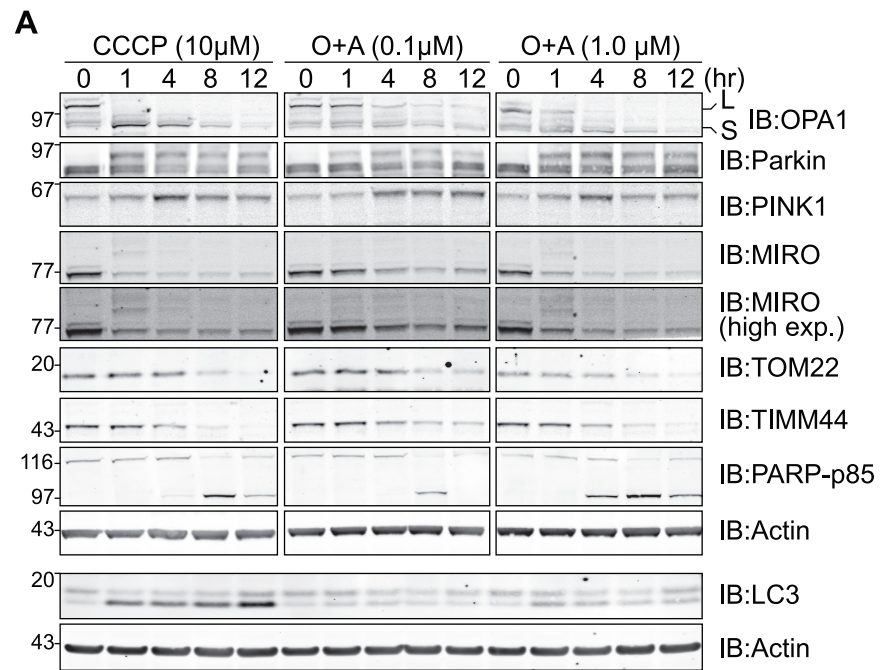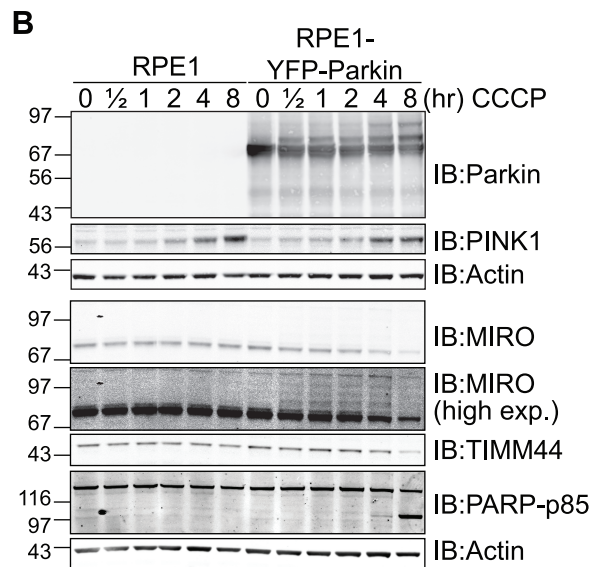

Supplement: Supplementary file 1 — Supplementary Figure S1 [file embr0016-0618-sd1.pdf]

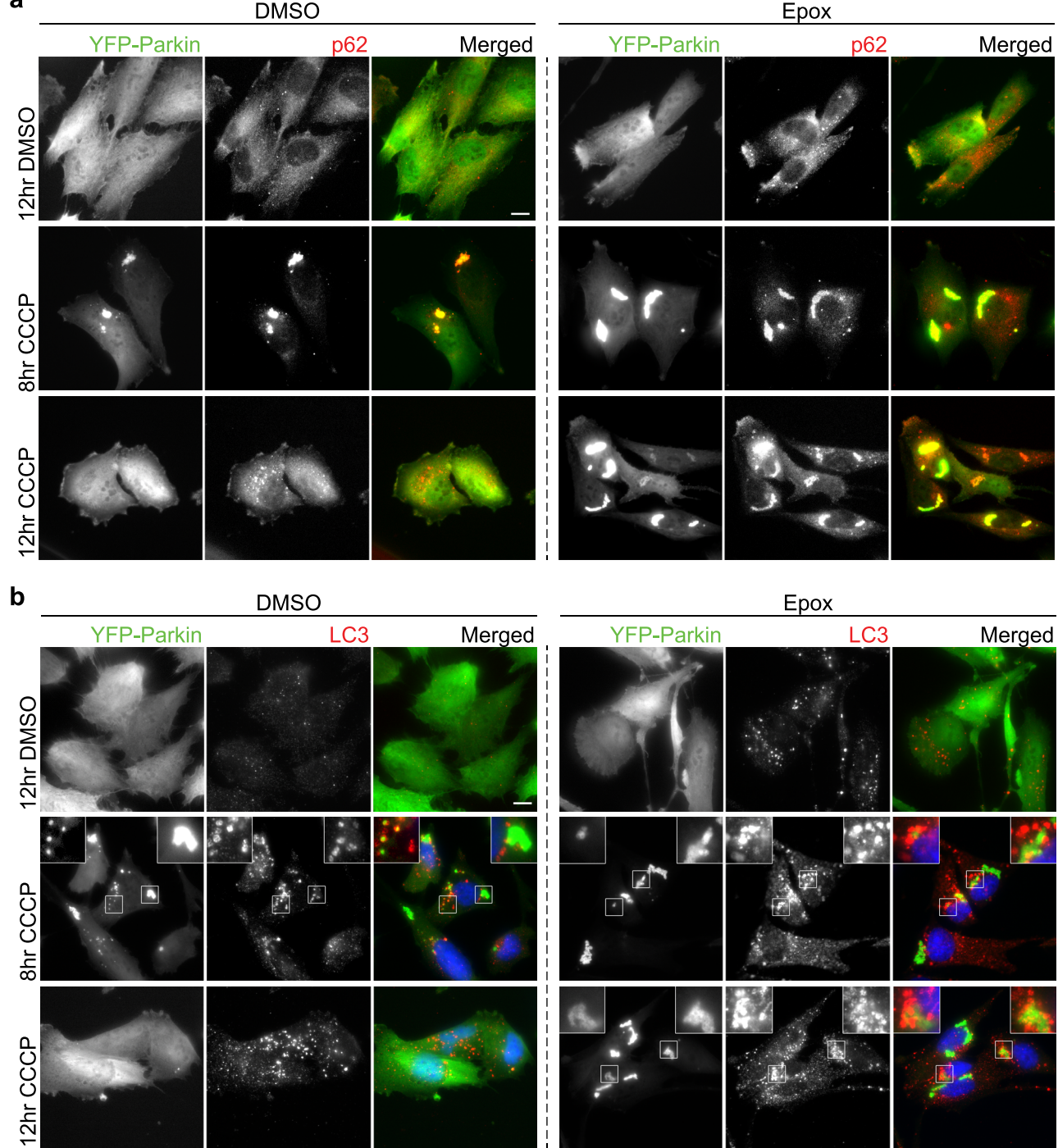

Liang et al. Supplementary Figure 2

Supplement: Supplementary file 2 — Supplementary Figure S2 [file embr0016-0618-sd2.pdf]

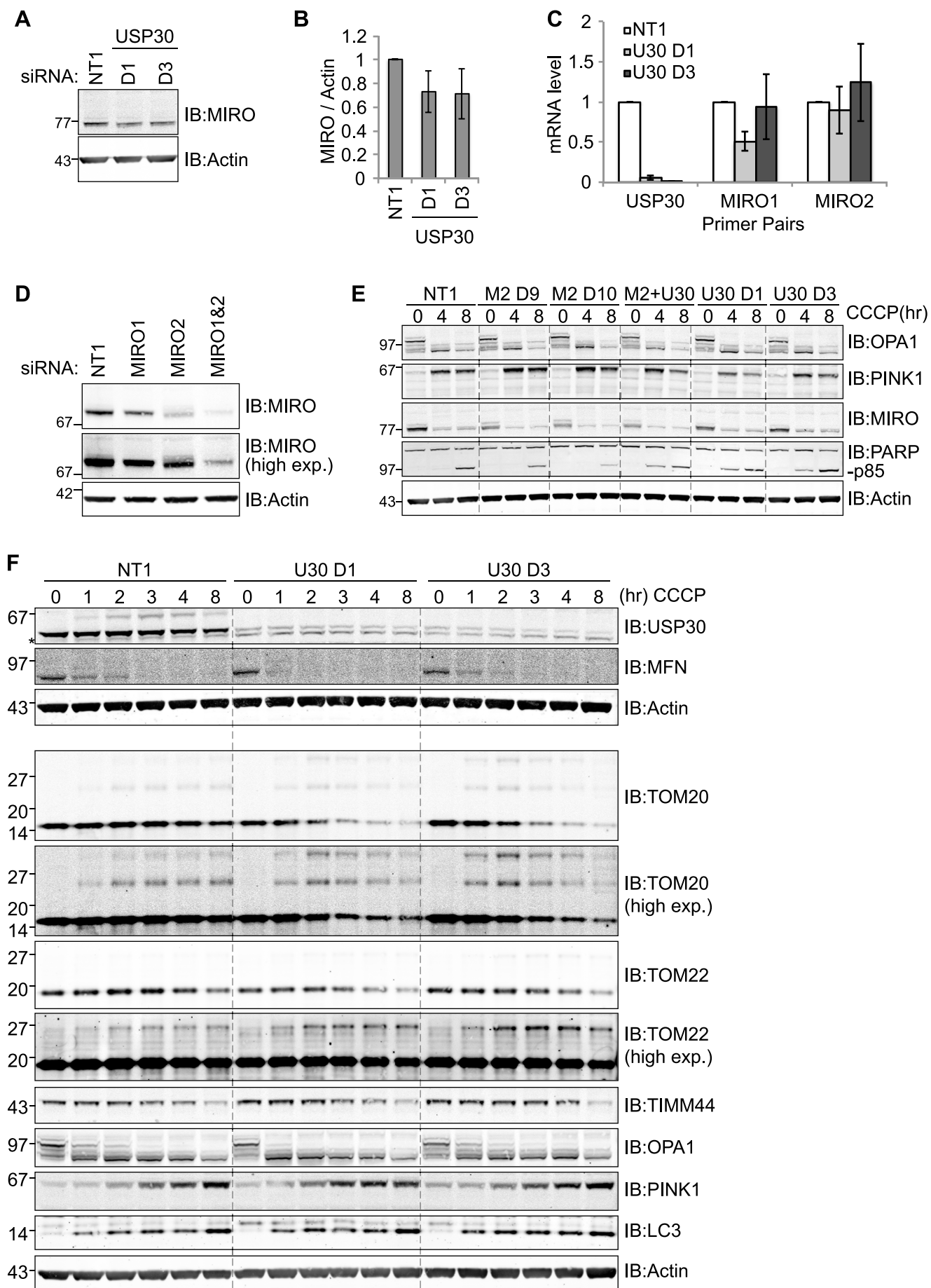

Liang et al. Supplementary Figure 3

Supplement: Supplementary file 3 — Supplementary Figure S3 [file embr0016-0618-sd3.pdf]

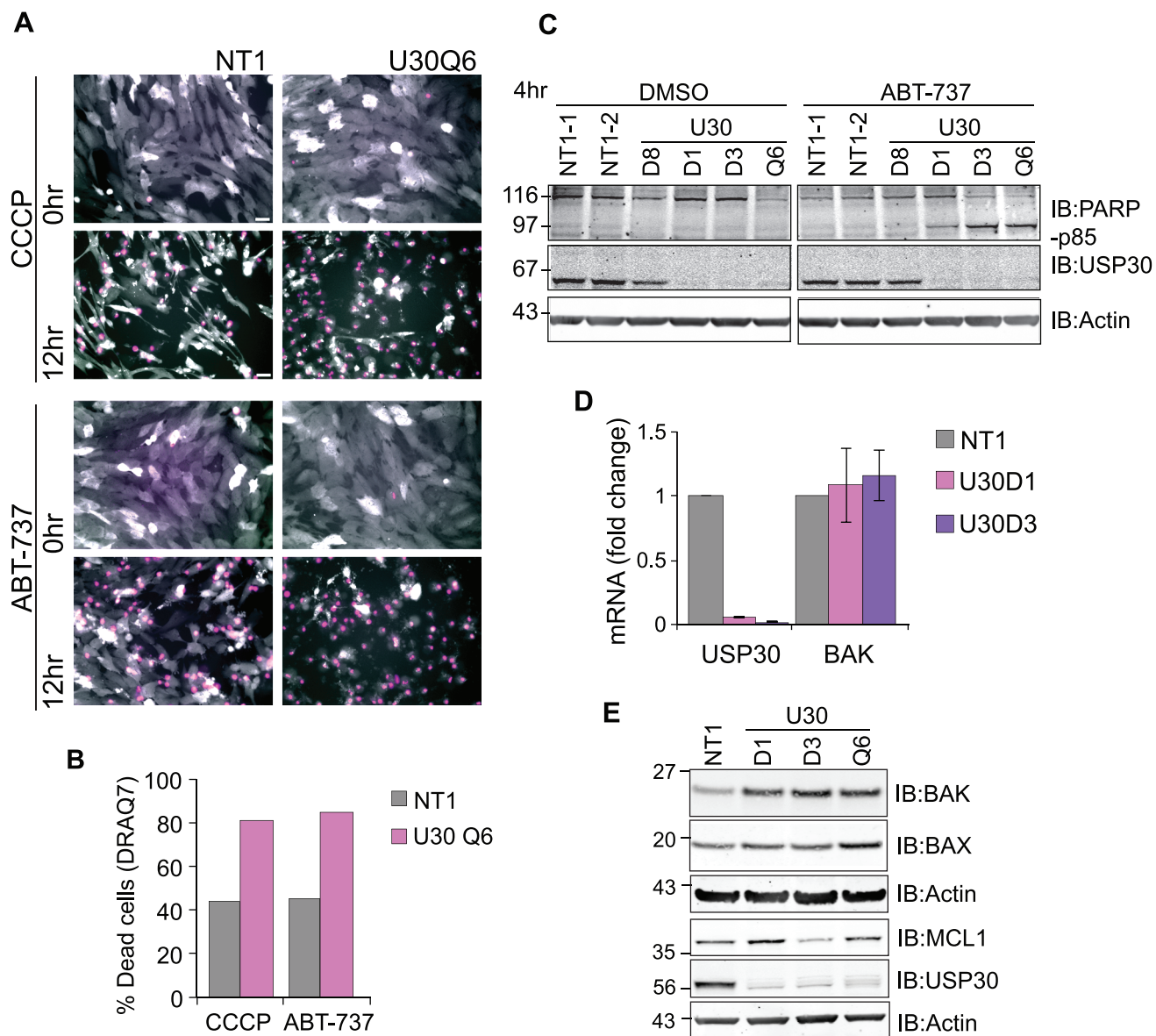

Liang et al. Supplementary Figure 4

Supplement: Supplementary file 4 — Supplementary Figure S4 [file embr0016-0618-sd4.pdf]

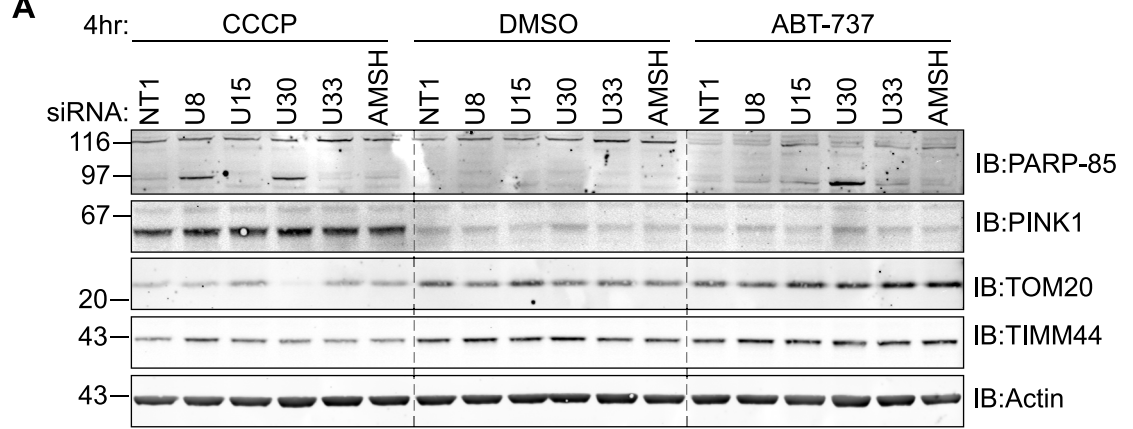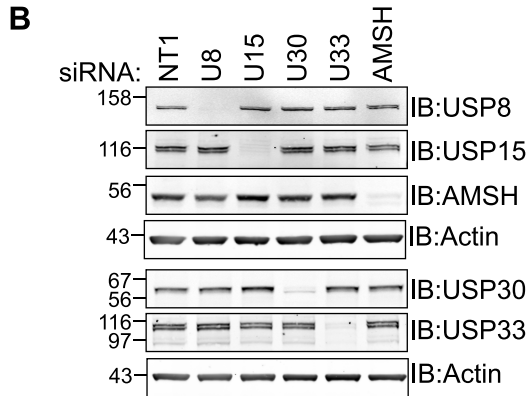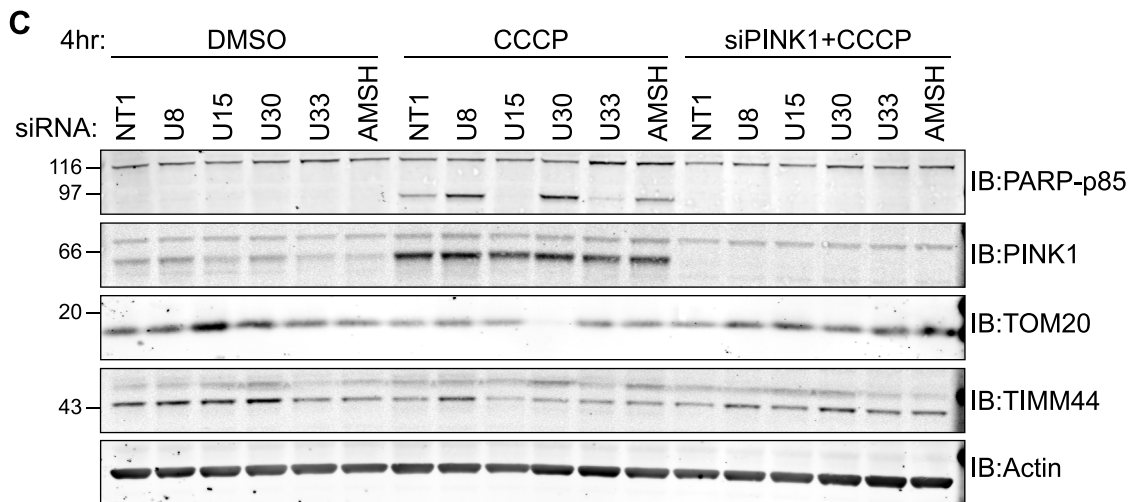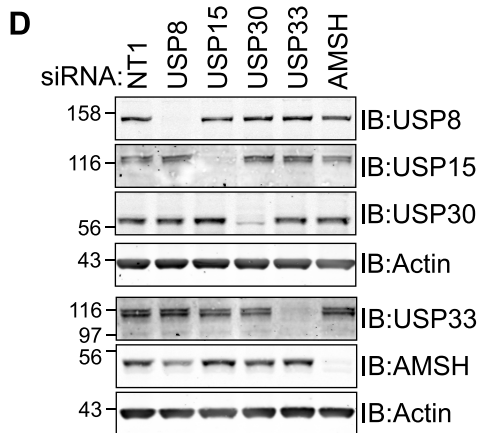

Supplement: Supplementary file 5 — Supplementary Figure S5 [file embr0016-0618-sd5.pdf]

Fig1A

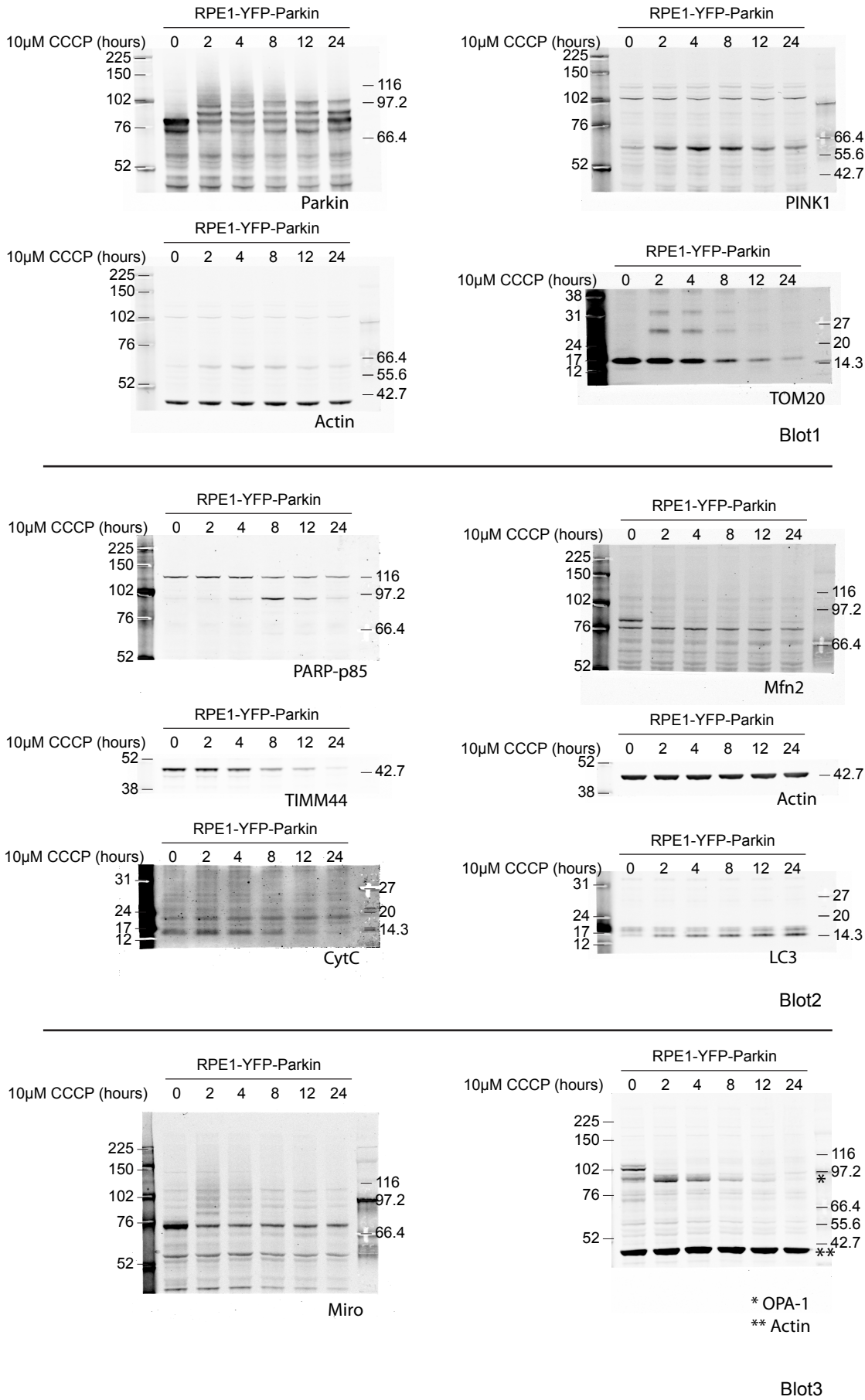

Fig 1B

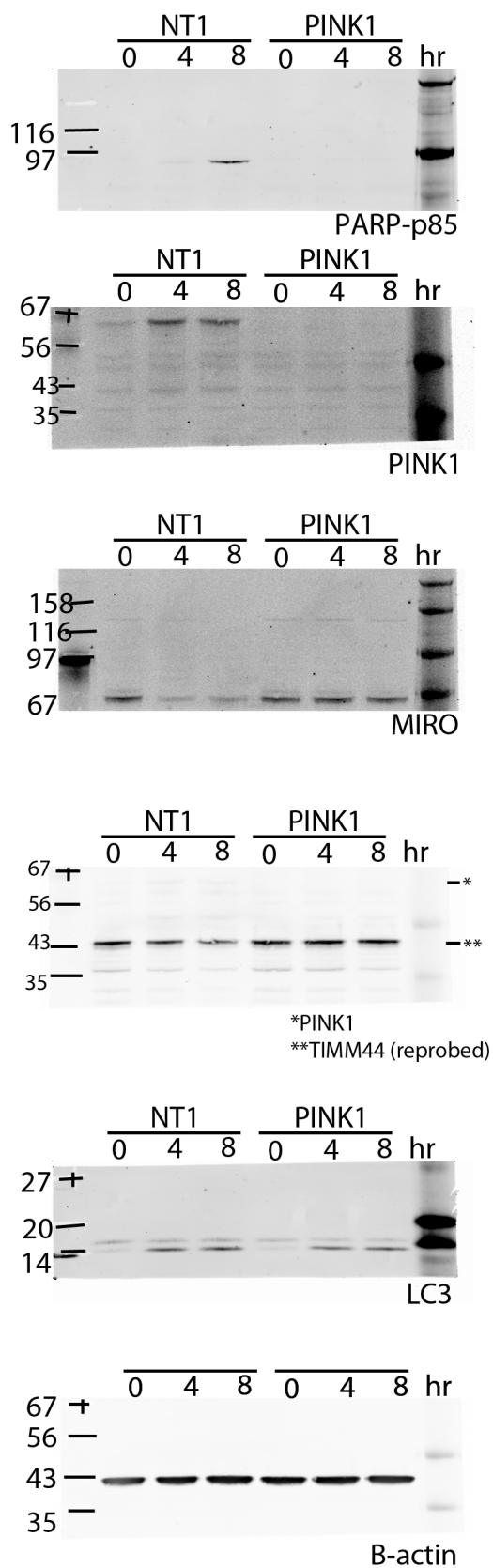

Fig 1F

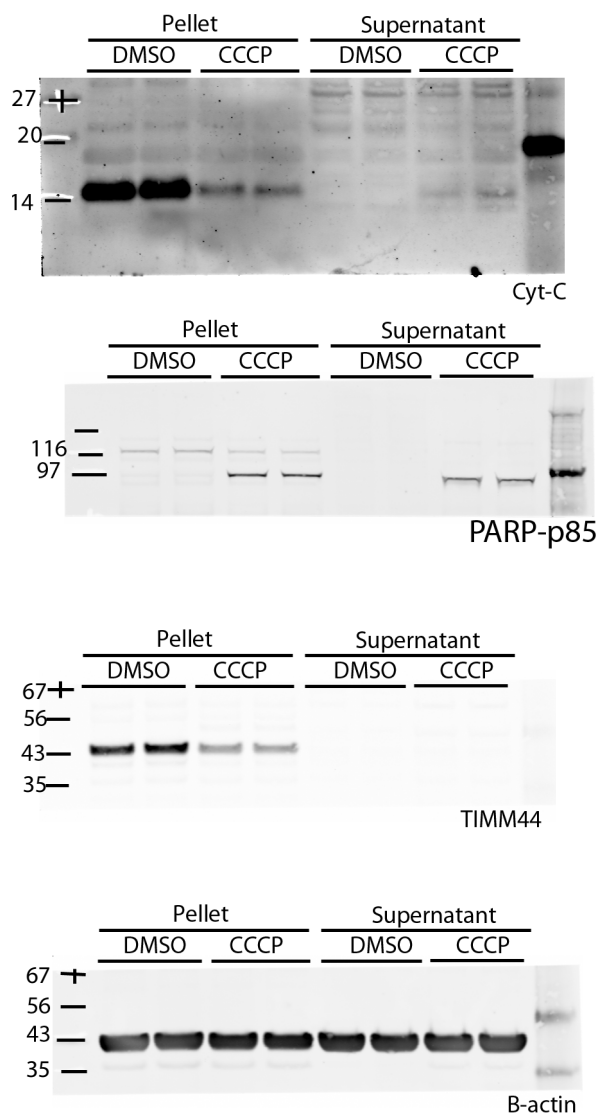

Fig 1G

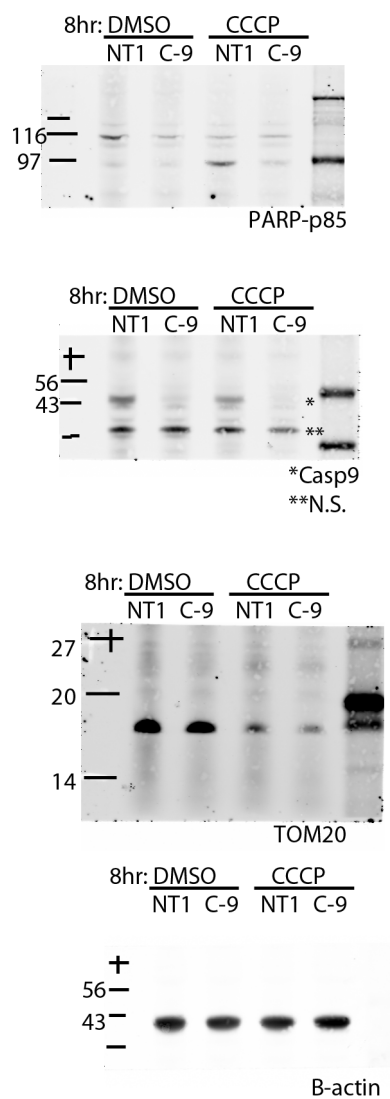

Fig 1H

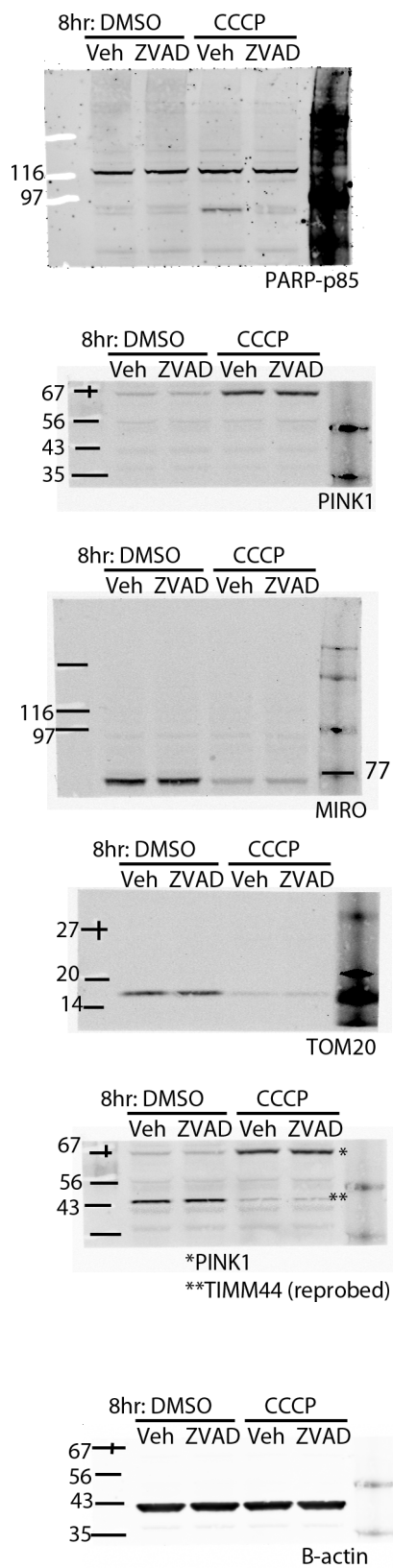

Supplement: Supplementary file 17 — Source Data for Figure 1 [file embr0016-0618-sd17.zip › source_data_figure1/source data figure 1.pdf]

Fig 2A

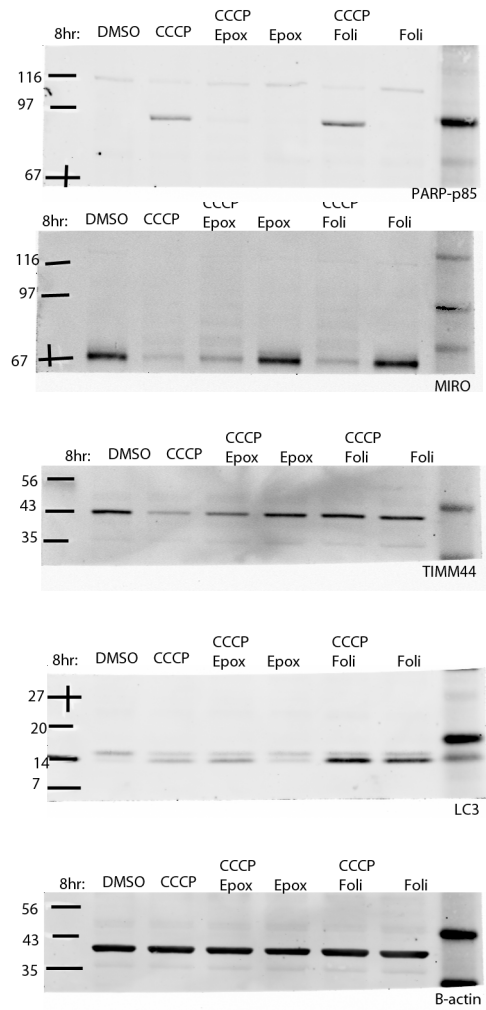

Fig 2C

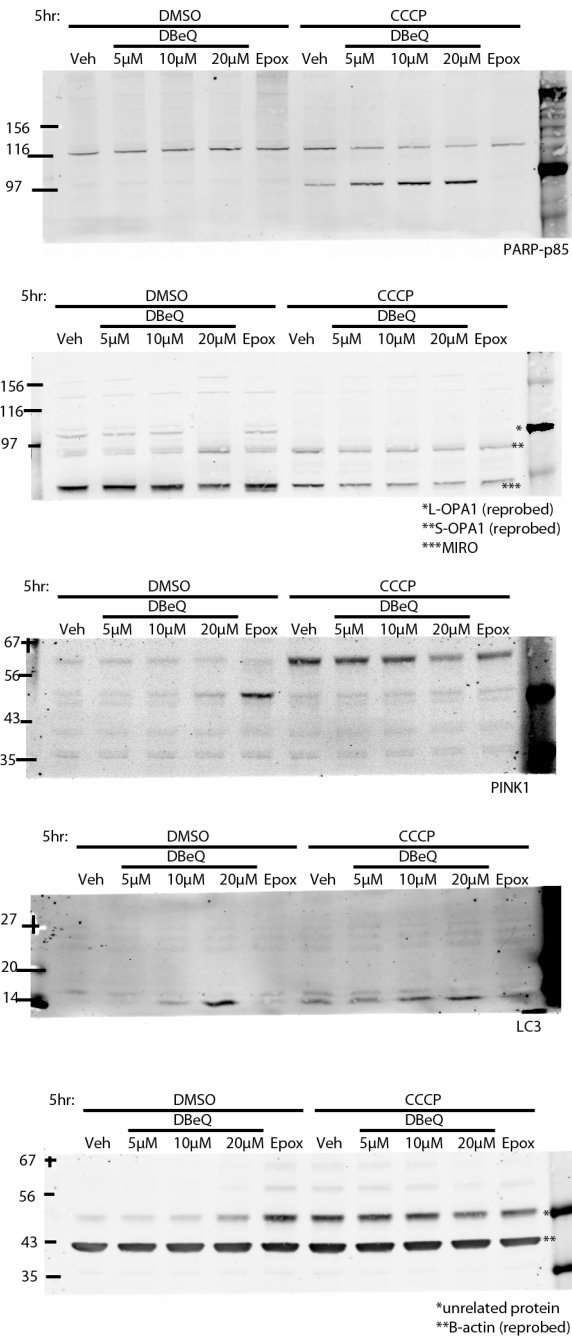

Supplement: Supplementary file 18 — Source Data for Figure 2 [file embr0016-0618-sd18.pdf]

Fig 3A

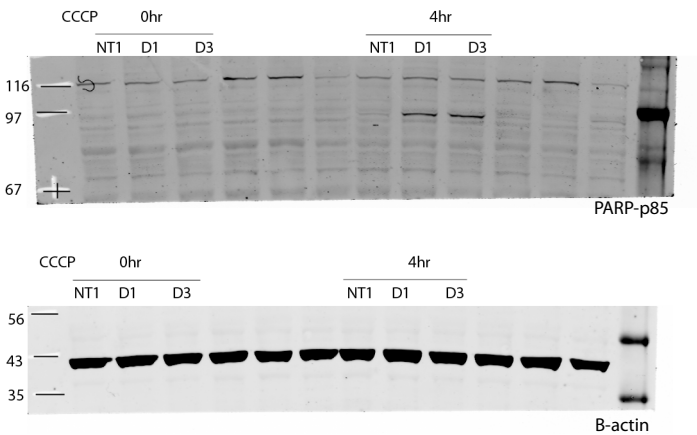

Fig 3C

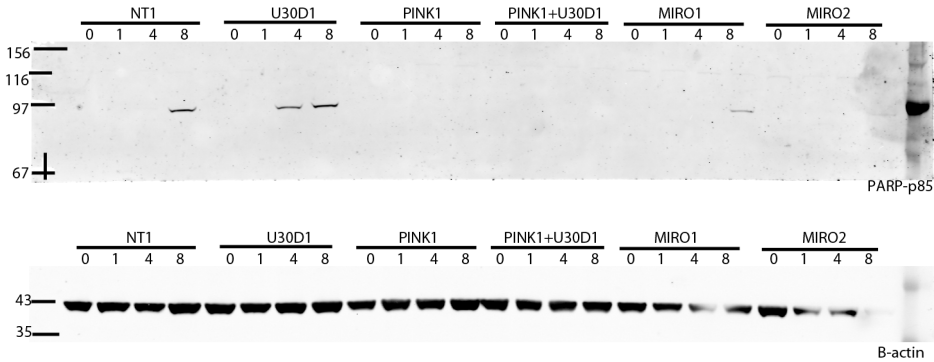

Fig 3D (part 1)

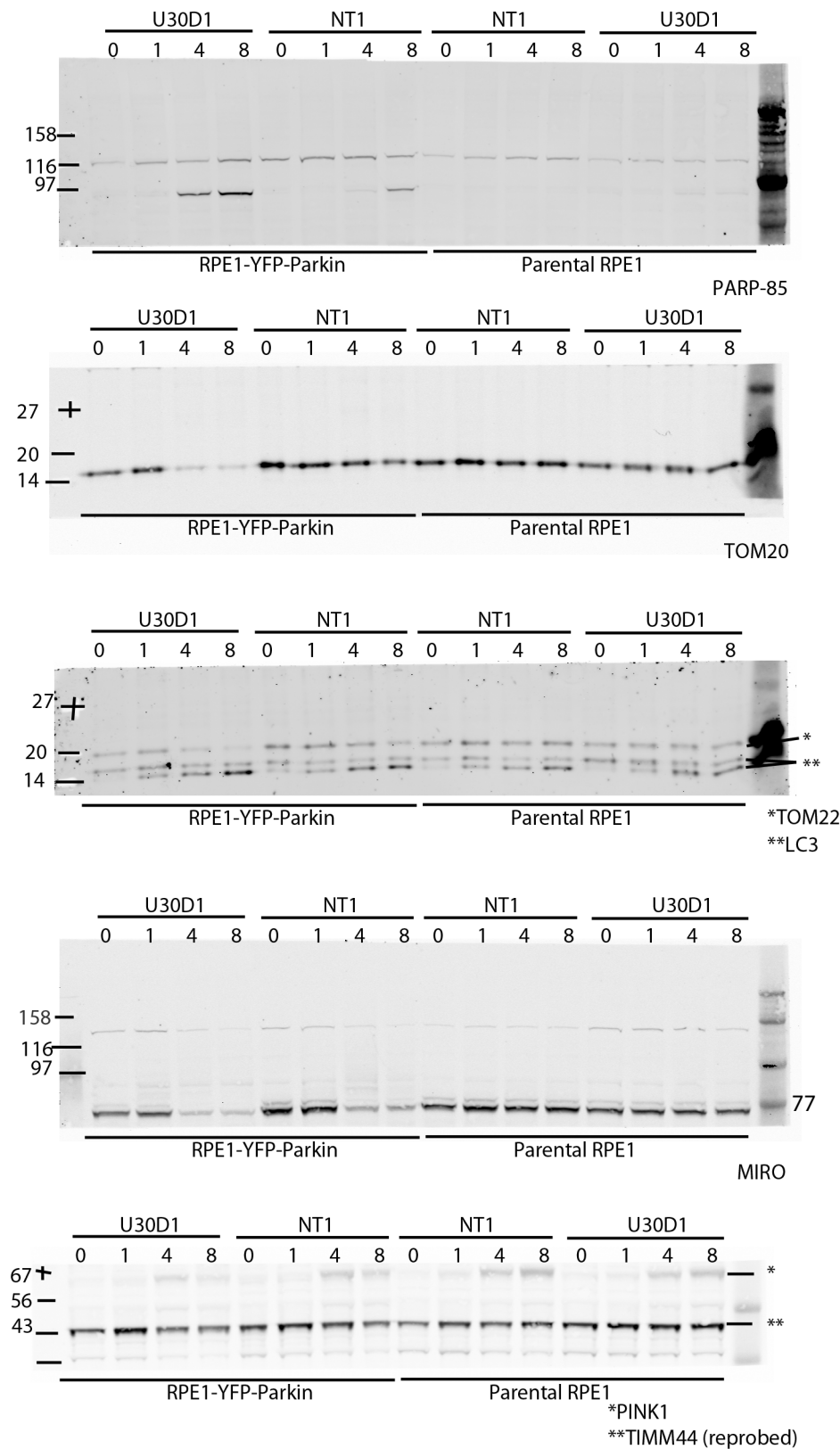

Fig 3D (part 2)

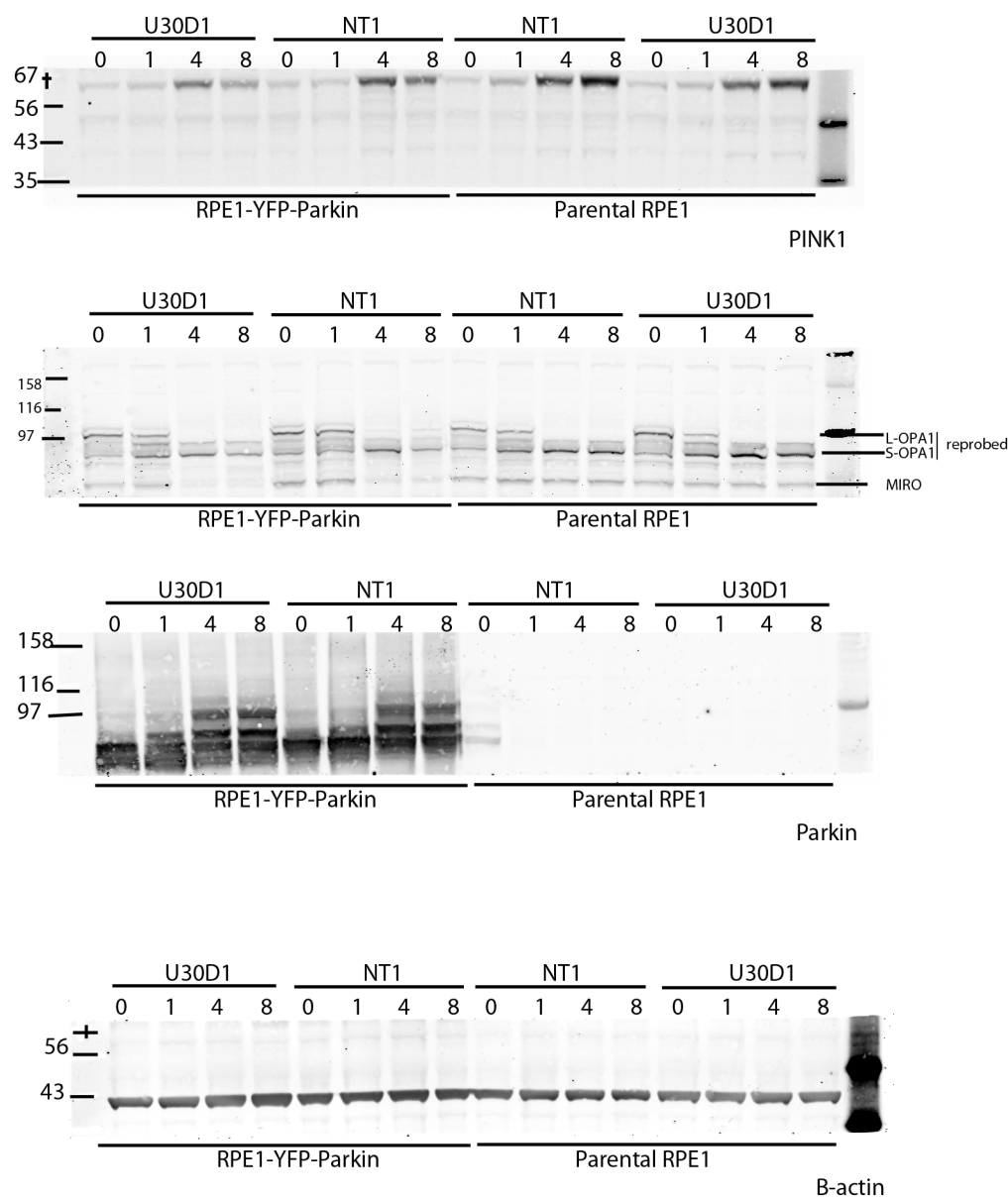

Fig 3E

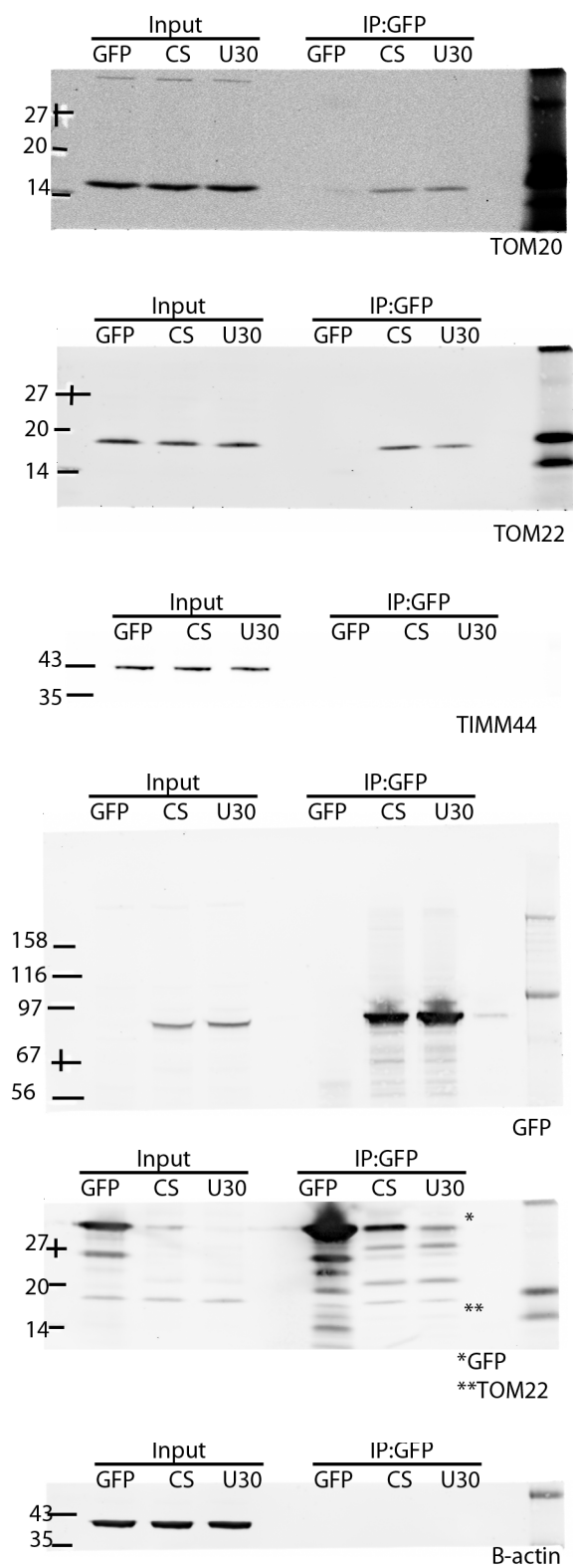

Fig 3F

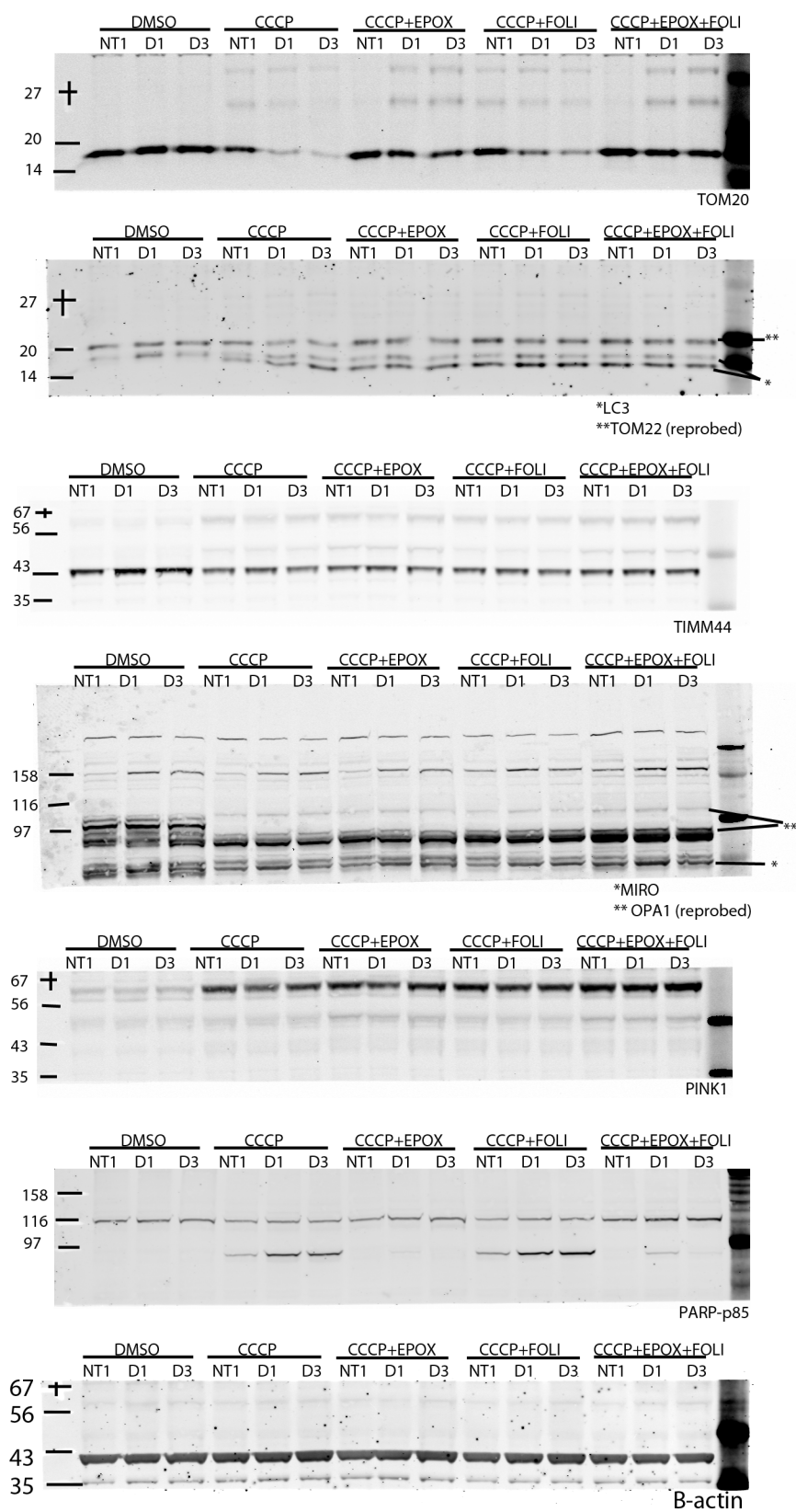

Supplement: Supplementary file 19 — Source Data for Figure 3 [file embr0016-0618-sd19.zip › Source_data_Figure3/Source_data_Figure3.pdf]

Fig 4C-part1

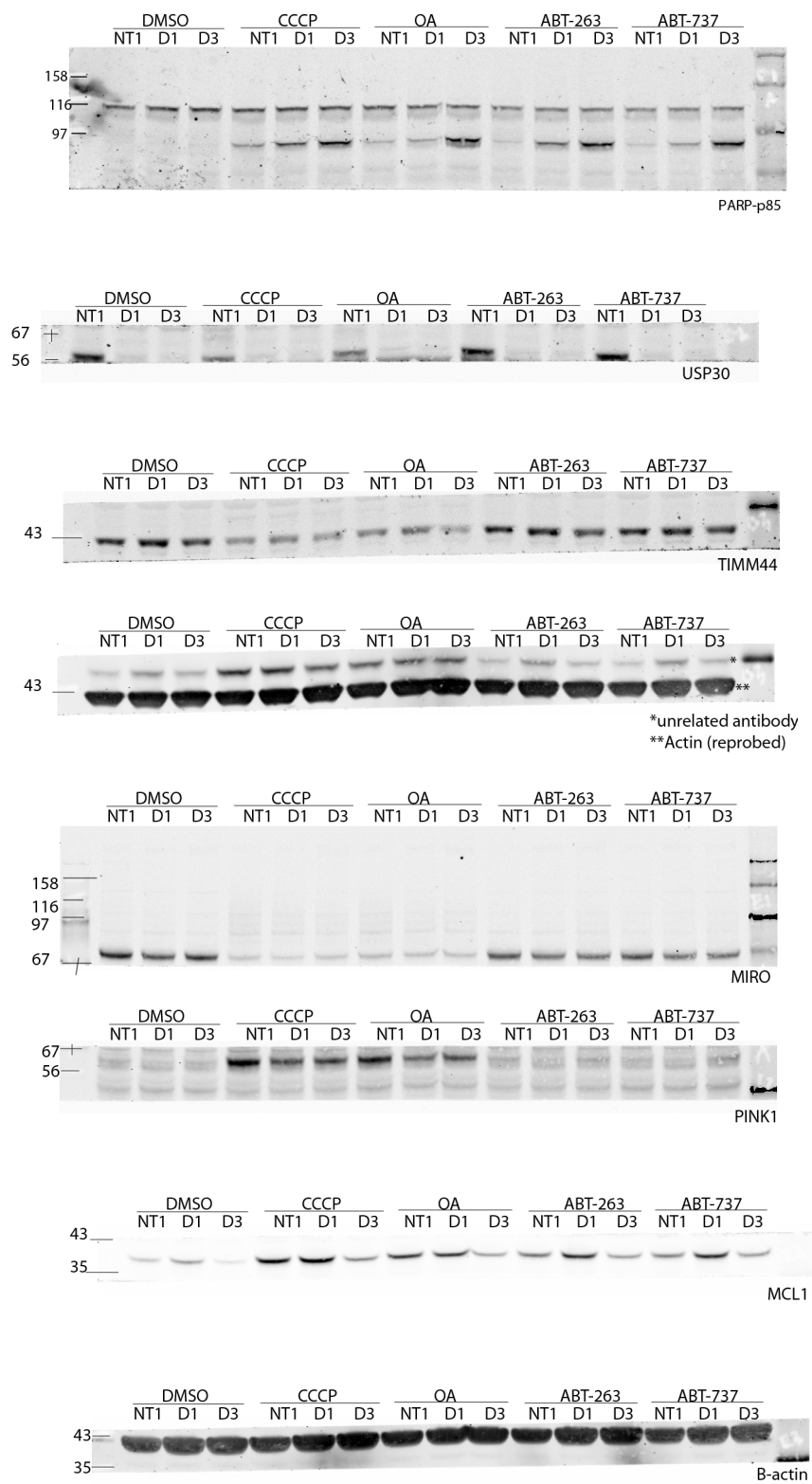

Fig 4C-part2

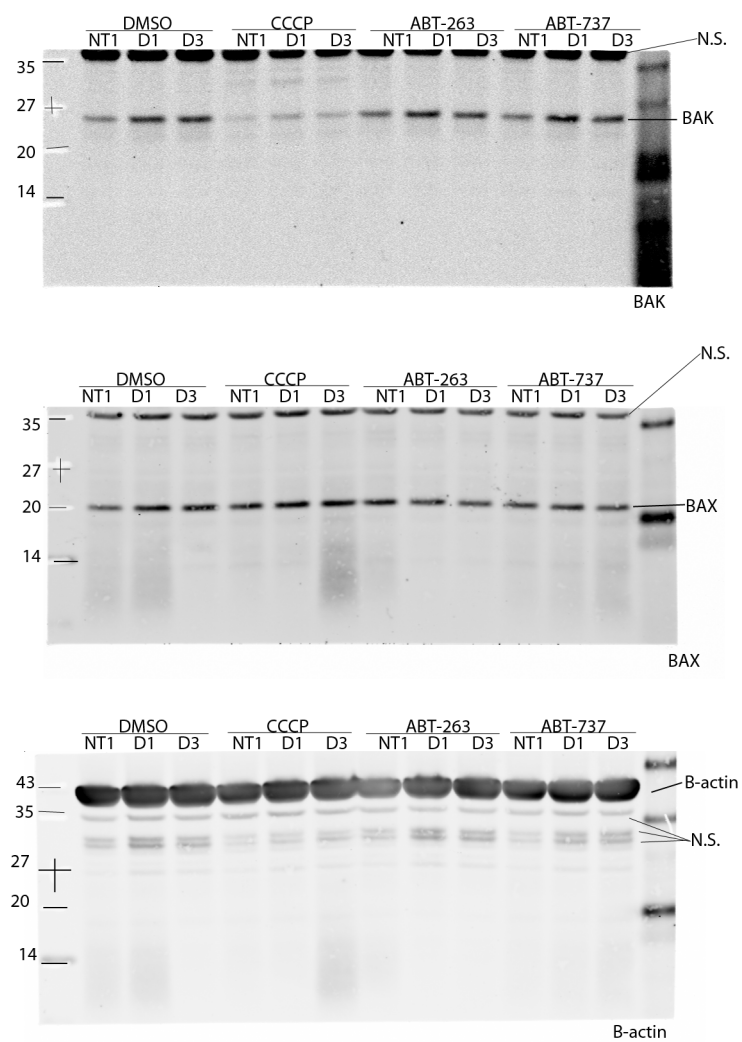

Fig 4D

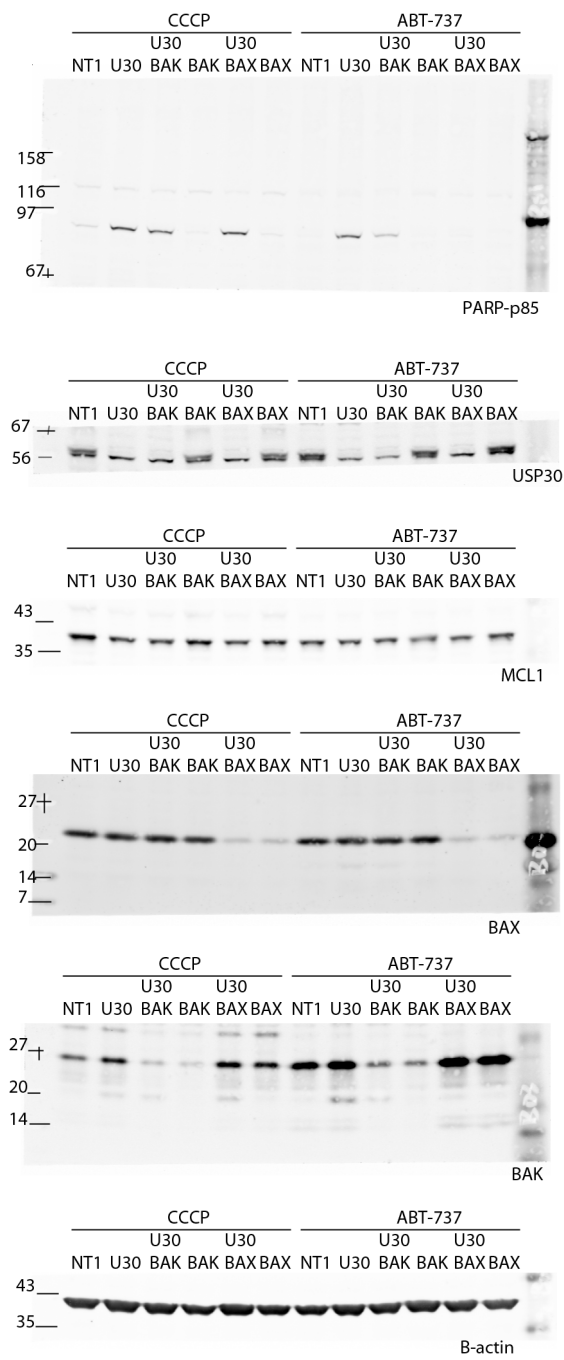

Supplement: Supplementary file 20 — Source Data for Figure 4 [file embr0016-0618-sd20.zip › Source_data_Figure4/Source_data_Figure4.pdf]

Fig 5A

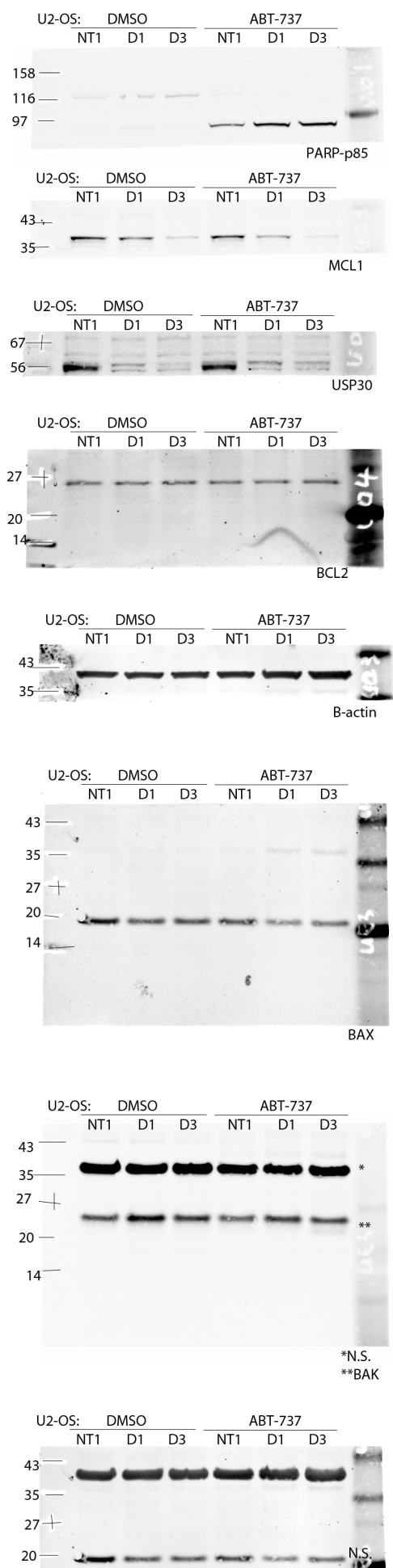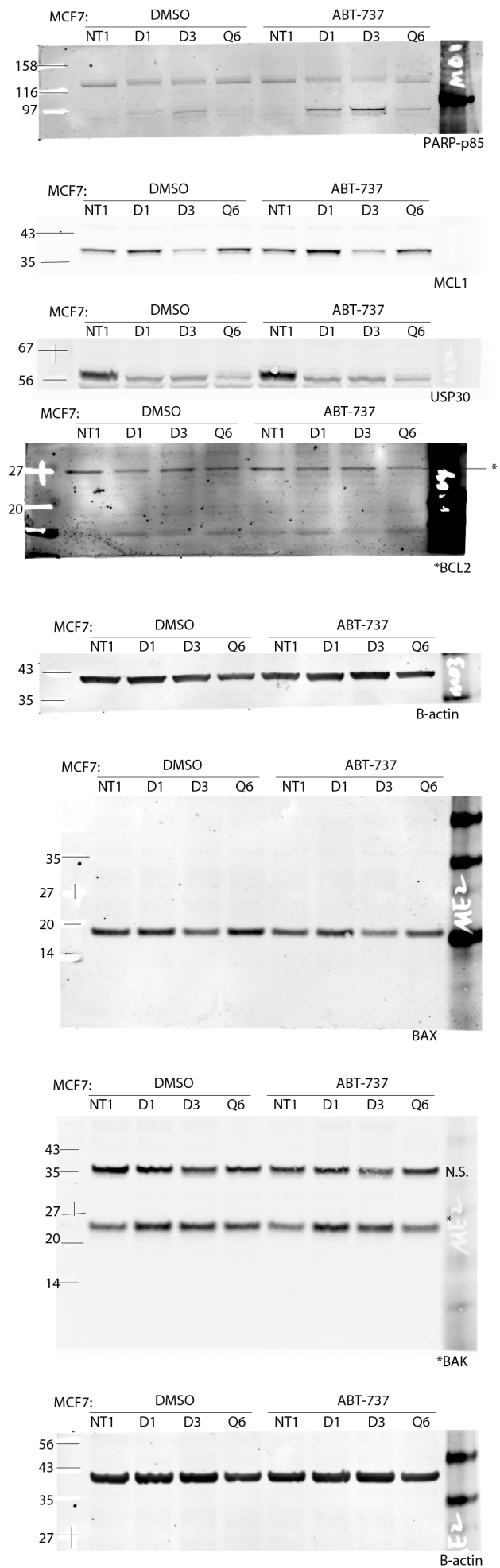

Supplement: Supplementary file 21 — Source Data for Figure 5 [file embr0016-0618-sd21.zip › Source_data_Figure5/Source_data_Figure5.pdf]
