# Supplementary material for: USP30 deubiquitylates mitochondrial Parkin substrates and restricts apoptotic cell death
Source: EMBO Rep. 2015 Mar 4;16(5):618–27. doi: 10.15252/embr.201439820 (PMC4428036; doi:10.15252/embr.201439820)
Supplement: Supplementary file 14 — Supplementary Methods [file embr0016-0618-sd14.pdf]

Supplementary Information: Methods

USP30 deubiquitylates mitochondrial Parkin substrates and opposes apoptotic cell death.

RNA interference and Plasmid transfection – For siRNA experiments, a reverse transfection protocol was performed using Lipofectamine RNAiMAX (Invitrogen) according to manufacturer's instruction with a seeding density per well of a 6-well plate of 3x10^5 cells for a 72hr siRNA experiment. Cells were transfected with the following siRNA oligos at 40nM final concentration: PINK1 (pool; 5'-GCAAAUGUGCUUCAUCUAA-3', 5'-GCUUUCGGCUGGAGGAGUA-3', 5'-GGACGCUGUUCCUCGUUAU-3' and 5'- GAGACCAUCUGCCCGAGUA-3'), Parkin (pool; 5'-GUAAAGAAGCGUACCAUGA-3', 5'-GAACAUCACUUCAUUACG-3', 5'-GAUAGUGUUUGUCAGGUUC-3' and 5'-UUAAAGAGCUCCAUCACUU-3'), BAK (pool; 5'-CGACAUGAACCGACGCUAU-3', 5'-UAUGAGUACUUCACCCCGA-3', 5'-GACGGCAGCUCGCCAUCAU-3' and 5'-AAUCAUGACUCCCAAGGGU-3') and BAX (pool; 5'-AAGUGGAGCUGACAUGUUU-3', 5-UGCCGGAACUGAUCAGAAC-3', 5'-GCAAACUGGUGCUCAAGGC-3' and 5'-CAUCAUGGGCUGGACAUUG-3'), ON-Target PLUS oligo pools (Dharmacon), USP30 siGENOME (D1, 5'-CAAAUUACCTGCCGCACAA-3' ; D3, 5'-ACAGGAUGCUCACGAAUUA-3'), USP30 On-Target Plus (D8, 5'-CGUCAGAUAUAAAGUCAUG-3'), MIRO1 ON-Target PLUS oligos (D9, 5'-UGUGGAGUGUUCAGCGAAAUU-3'; D10, 5'-GCAAUUAGCAGAGGCGUUAUU-3'; D11, 5'-CCAGAGAGGGAGACACGAAUU-3'; D12, 5'-GCUUAAUCGUAGCUGCAAAUU-3'), MIRO2 ON-Target PLUS oligos (D9, 5'-GAGUGGAGUGUUCGGCCAAUU-3'; D10, 5'-CCUCAAGUUUGGAGCCGUUUU-3'; D11, 5'-GAGGUUGGGUCCCUGAUUAUU; D12, AGGAGAUCCACAAGGCAAAUU-3'), USP8 (D1, 5'-UGAAAUACGUGACUGUUUAUU-3'), USP15 (D17, CAG ATA AGG TGG TTG CCG A), USP33 (D6, UGGGCAUGUCUGGAGAAUAGA), AMSH (D2, 5'-UUACAAAUCUGCUGUCAUUUU-3'). Control samples were treated with ON-TARGETplus Non-Targeting siRNA oligo #1 (NT1; 5'-UGGUUUACAUGTCGACUAA-3'). Caspase 9 (SI02654610; 5'-GUGACAUCUUUGUGUCCUAUU-3') and USP30 (Q6, 5'- CUCCGAUGACACUGUCCGCAA-3') siRNA oligos were from Qiagen. Medium was exchanged the following day after transfection. For 24hr overexpression in HEK293T cells, 1μg and 3μg of plasmid were used per well of a 6-well plate and

per 10cm dish respectively, at a ratio of 1:3 (μg/μl) of plasmid to Genejuice (Novagen).

Cell lysis and Western blots- Cells were harvested with either NP-40 lysis buffer (0.5% w/v Nonidet P-40, 25mM Tris-HCl pH7.5, 100mM NaCl, 50mM NaF, Figures 1B, 2A, 3A, 3E, 4C, 4D), RIPA lysis buffer (1% Nonidet P-40, 10mM Tris-HCl, pH7.5, 150mM NaCl, 1% sodium deoxycholate, 0.5% SDS, Figures 1H, 2C, 3C, 3D, 3F), supplemented with mammalian protease inhibitor cocktail (Sigma) or 'hot lysis buffer' (1% SDS, 50mM NaF and 1mM EDTA at 110°C, Figures 1A, 1G, 4B). In some experiments, PhosSTOP (Roche) was also added. Lysates were precleared by centrifugation at 14,000 x g. Protein concentrations were determined using BCA protein assay (Pierce) and equal amounts of protein were analyses by SDS-PAGE and transferred to nitrocellulose membrane. Western blots were scanned using the Licor Odyssey system (LI-COR Biosciences) and the images analyzed using ImageJ.

Digitonin fractionation- Cells treated with DMSO or CCCP were scraped off the dish in ice-cold PBS using a rubber policeman, spun down at 700 x g for 5min and resuspended in 200μl of HIM buffer (200mM D-mannitol, 70mM sucrose, 1mM EGTA, 10mM HEPES, pH7.5) with 0.025% digitonin and mammalian protease inhibitor. The cell suspension was incubated on ice for 10min and centrifuged at 20,000 x g for 5min. The pellet was then resuspended in 200μl of RIPA lysis buffer supplemented with mammalian protease inhibitor and pellets and supernatants analysed side by side by SDS-Page and subsequent western blotting.

Immunoprecipitation- Precleared lysates were incubated with anti-GFP and protein G agarose for 2hr at 4°C. Beads were washed three times with YP-IP buffer (0.1% Nonidet P-40, 25mM Tris/HCl, pH7.5, 150mM NaCl) and once with 10mM Tris/HCl, pH7.5 and resuspended in 1.5 x SDS-PAGE sample buffer.

Antibodies- Rabbit anti-OPA1 (ab5404) and mouse anti-Actin (ab6276), anti-BCL2 (ab692) were obtained from Abcam (Cambridge, MA). Mouse anti-PARP-p85 (9546) and rabbit anti-BCL-XL (2764) were purchased from Cell Signaling Technologies (Danvers, MA), mouse anti-p62 (610833) and mouse anti-Cytochrome C (556432, IF), anti-BAX (610982) from BD Transduction Laboratories, and mouse anti-LC3 (5F10) from Nanotools. Rabbit anti-PINK1 (BS100-494) was obtained from Novus

Biologicals, mouse anti-Parkin (sc32282), rabbit anti-BAK (sc832), anti-MCL-1 (sc819) were from Santa Cruz and mouse anti-TOM22 (1C9-2; T6319), anti-Tubulin (T5168), rabbit anti-mitofusin 2 (M6444), anti-Actin (A2206), anti-TIMM44 (HPA043052), anti-MIRO1 (HPA010687), anti-TOM20 (HPA011562), mouse anti-USP33 (WH0023032M1) and sheep anti-Cytochrome C (C5723) were purchased from Sigma-Aldrich. Rabbit anti-USP8 was purchased from Bethyl (A302-929A), and mouse anti-USP15 from Abnova (H00009958-M01). Sheep anti-GFP was a gift from Ian Prior (University of Liverpool, United Kingdom). Rabbit anti-AMSH has previously been described in McCullough et al [1]. Rabbit anti-USP30 was a generous gift from Baris Bingol (Genentech, US). Secondary IR680- and IR800-coupled donkey anti-mouse, anti-rabbit and anti-sheep were from Licor Biosciences.

RNA Extraction and Real-time PCR - Cultured RPE1-YFP-Parkin cells were harvested 24hr or 72hr post-transfection and total RNA was extracted using RNeasy columns (Qiagen). cDNA was reverse-transcribed from 1μg of RNA with RevertAid H-minus M-MuLV reverse transcriptase (Fermentas) using an oligo(dT) primer (Promega). Quantitative real-time RT-PCR (qRT-PCR) was performed in triplicates using SYBR Green Supermix and the CFX Connect Real-Time PCR Detection System (Bio-Rad). Primer sequences used for qRT-PCR to amplify mRNA were as follow: USP30 [forward: 5'-GCACCTTCTGAAAGCCTTGT-3'; reverse: 5'-CGTGAGCATCCTGTTCTTCA-3'], MIRO1 [forward: 5'-ATCCAGAGAGGGAGACACGA-3'; reverse: 5'-GCTCATCAGGTGACAAAGCA-3'], MIRO2 [forward: 5'-AGTGGGCAACAAGTCAGACC-3'; reverse: 5'-GCTTGGCCTCAGGGTCATAG-3'], BAK [forward: 5'- ATTGCCACCAGCCTGTTTGA-3'; reverse: 5'- ATGCAGCATGAAGTCGACCA-3']. Samples were analyzed with 2-step amplification and melt curves were analysed after 40 cycles. The Ct value for test genes were normalized to beta-Actin and the relative expression of each gene was represented as $2^{-[\Delta\Delta Ct]}$.

Quantitation of live cell imaging experiments:

Three to four independent fields (xy positions) were chosen at random for image acquisition and sample sizes were kept similar between experimental groups. A total number of 280 - 900 total cells were recorded per sample per experiment. The total number of dead cells was determined by counting DRAQ7-positive cells at 12 or 6

hours or AnnexinV-positive cells at 6 hrs after application of CCCP or ABT-737 respectively, and compared to the total number of cells at 0hrs.

P values are indicated as *P,0.05, **P,0.01 and ***P,0.001 and were derived either by two tailed paired t-test or, for multiple comparison analysis, by one-way ANOVA and Dunnett's post-hoc test using GraphPad Prism6.

Reference

1. McCullough J, Clague MJ, Urbe S (2004) AMSH is an endosome-associated ubiquitin isopeptidase. *J Cell Biol* **166:** 487-492
