# Supplementary material for: USP30 deubiquitylates mitochondrial Parkin substrates and restricts apoptotic cell death
Source: EMBO Rep. 2015 Mar 4;16(5):618–27. doi: 10.15252/embr.201439820 (PMC4428036; doi:10.15252/embr.201439820)
Supplement: Supplementary file 15 — Supplementary Legends [file embr0016-0618-sd15.doc]

**Supplementary Information – Figure legends**

**Figure S1. Parkin-dependent mitophagy and associated cell death.**

A hTERT-RPE1-YFP-Parkin cells were treated with either 10µM of CCCP or oligomycin A and antimycin A (0.1µM each or 1µM each) for the indicated times. The cells were harvested with NP40 lysis buffer supplemented with mammalian protease inhibitor. L, long form of OPA1; S, short form of OPA1.

B hTERT-RPE1 parental cells and hTERT-RPE1-YFP-Parkin cells were treated with CCCP (10µM) for 0, 0.5, 1, 2, 4, 8hrs. Arrow indicates endogenous Parkin. high exp: high exposure.

**Figure S2. Proteasome inhibitors delay Parkin-mediated mitophagy.**

A hTERT-RPE1-YFP-Parkin cells were treated with 10µM of CCCP and 100nM of epoxomicin for 8 and 12hr. The cells were fixed with PFA and stained with anti-p62 antibody. Scale bar: 10 µm.

B hTERT-RPE1-YFP-Parkin cells were treated as in (A), then fixed with PFA and methanol and stained with anti-LC3 antibody. Insets show 3-fold enlargement of the boxed area.

**Figure S3. USP30 depletion decreases basal MIRO levels and promotes the degradation of TOM20.**

A hTERT-RPE1-YFP-Parkin cells were transfected with two individual USP30-targeting siRNA oligos (40nM, D1 and D3) for 72hr. Cells were lysed in NP40 lysis buffer and probed as indicated.

B The band intensity of MIRO (normalised to actin) is expressed as a mean ±SD of 5 independent experiments.

C hTERT-RPE1-YFP-Parkin cells were transfected as in A and harvested 24hr after transfection for mRNA extraction and processing for qRT-PCR. USP30, MIRO1 and MIRO2 mRNA was normalized to Actin mRNA (n=3 independent experiments, error bar shows SD)

D hTERT-RPE1-YFP-Parkin cells were transfected with siRNA (40nM) against MIRO1 and MIRO2 for 72hr. Cells were lysed in NP40 lysis buffer and probed with the indicated antibodies.

E hTERT-RPE1-YFP-Parkin cells were transfected with siRNA oligos targeting MIRO2 (M2, D9 and D10), or USP30 (D1 and D3), or with siRNAs targeting both MIRO2 (D9 and D10) and USP30 (D1and D3), treated with CCCP and harvested in RIPA lysis buffer supplemented with mammalian protease inhibitor at indicated times. F hTERT-RPE1-YFP-Parkin cells were transfected for 72 hrs with either non-targeting siRNA (NT1) or USP30-targeting siRNA (D1 and D3, 40 nM). Cells were treated with 10µM of CCCP for the depicted time and harvested using RIPA lysis buffer supplemented with mammalian protease inhibitor and NEM**.** Asterisk indicates a non-specific band.

**Figure S4 – Additional siRNA oligo and BAK mRNA levels.**

A hTERT-RPE1-YFP-Parkin cells were transfected for 72 hrs with NT1 or USP30 siRNA oligo Q6, then treated with CCCP or ABT-737 (10 µM) and imaged every 15 min in the presence of DRAQ7 (see Movie 6).

B Graph shows % of DRAQ7 positive cells for cells treated as in A after 12 hours of treatment for a representative experiment, cells counted in four independent positions (CCCP: NT1 – total 507, dead 221, 43.5% SD± 4.2; USP30 Q6 – total 398, dead 323, 81.2% SD±1.8; ABT-7373: NT1 – total 646, dead 293, 45.4% SD±8.3; USP30 Q6 – total 366, dead 311, 85% SD±9.9), see Movie 6.

C hTERT-RPE1-YFP-Parkin cells were transfected for 72 hrs with NT1 (NT1-1 and NT1-2: duplicate samples) or USP30 targeting siRNA (D1, D3, D8 and Q6), then treated with ABT-737 (all 10 µM) for 4 hrs, harvested and probed as indicated.

D hTERT-RPE1-YFP-Parkin cells were transfected as in A and harvested 72hr after transfection for mRNA extraction and processing for qRT-PCR. USP30 and BAK mRNA were normalized to Actin mRNA (n=3 independent experiments, error bar shows SD).

E hTERT-RPE1 cells were transfected for 72 hrs with NT1 or USP30 targeting siRNA (D1, D3 and Q6), then lysed and probed as indicated. Note that this is an expanded view of the DMSO arm of the experimental data shown in Figure 5A.

**Figure S5 – Comparison with other DUBs implicated in mitophagy and the autophagic-lysosomal pathways.**

A hTERT-RPE1-YFP-Parkin cells were transfected with 40nM of siRNA against NT1, USP8 (D1), USP15 (D17), USP30 (D3), USP33 (D6), AMSH (D2) for 72hrs. Cells were then treated, with either DMSO, CCCP (10µM) or ABT-737 (10µM) for 4hrs, harvested in NP40 lysis buffer and lysates were probed as indicated.

B DMSO treated samples shown in A were probed as indicated.

C, D hTERT-RPE1-YFP-Parkin cells were transfected with 40nM of siRNA against NT1, PINK1 (pool), USP8 (D1), USP15 (D17), USP30 (D3), USP33 (D6), AMSH (D2) for 72hrs. Cells were then treated as in A and probed as indicated.

D DMSO treated samples shown in C were probed as indicated.

MOVIES

**Movie 1.** **Depletion of PINK1 protects Parkin-overexpressing cells from CCCP-induced cell death.** hTERT-RPE1-YFP-Parkin cells were transfected for 72 hours with either non-targeting (NT1) or PINK1-targeting siRNA (40 nM). Cells were treated with CCCP (10µM) and imaged in the presence of the membrane impermeable dye DRAQ7 (pink). YFP-Parkin is shown in grey. Frames were collected every 30 min and saved for playback at 3 frames/sec.

**Movie 2.** **Proteasome inhibition delays CCCP-induced cell death.** hTERT-RPE1-YFP-Parkin cells were transfected for 72 hours with non-targeting (NT1, 40 nM) and treated with CCCP (10µM) in the absence or presence of Epoxomycin (100 nM) and imaged in the presence of the membrane impermeable dye DRAQ7 (pink). YFP-Parkin is shown in grey. Frames were collected every 30 min and saved for playback at 3 frames/sec.

**Movie 3.** **siRNA-mediated depletion of USP30 enhances CCCP-induced cell death.** hTERT-RPE1-YFP-Parkin cells were transfected for 72 hrs with either non-targeting siRNA (NT1, see also Supplementary Movie 2) or USP30-targeting siRNA (D1 and D3, 40 nM). Cells were treated with CCCP (10µM) and imaged in the presence of the membrane impermeable dye DRAQ7 (pink). YFP-Parkin is shown in grey. Frames were collected at 30 min intervals and saved for playback at 3 frames/sec.

**Movie 4.** **USP30 depletion sensitizes cells to cell death induced by the BH3-mimetic ABT-263.** hTERT-RPE1-YFP-Parkin cells were transfected for 72 hrs with either non-targeting siRNA (NT1) or USP30-targeting siRNA (D1 and D3, 40 nM). Cells were then treated with ABT-263 (10µM) either on its own or in combination with Epoxomicin (100nM, bottom left panel). Cells were imaged in the presence of the membrane impermeable dye DRAQ7 (pink). YFP-Parkin is shown in grey. Frames were collected at 30 min intervals and saved for playback at 3 frames/sec.

**Movie 5.** **USP30 depletion sensitizes Parkin-overexpressing cells to cell death induced by the BH3-mimetic ABT-737.** hTERT-RPE1-YFP-Parkin cells were transfected for 72 hrs with either non-targeting siRNA (NT1, top and bottom left panels, or siRNA targeting USP30 (D1 and D3). Cells were then treated with ABT-737 (10µM) and imaged in the presence of the membrane impermeable dye DRAQ7 (pink). YFP-Parkin is shown in grey. Frames were collected at 30 min intervals and saved for playback at 3 frames/sec.

**Movie 6.** **USP30 depletion sensitizes Parkin-overexpressing cells to cell death induced by the BH3-mimetic ABT-737– additional siRNA oligo (Q6).** hTERT-RPE1-YFP-Parkin cells were transfected for 72 hrs with either non-targeting siRNA (NT1, top and bottom left panels, or siRNA targeting USP30 (D1 and D3, Q6). Cells were then treated with ABT-737 (10µM) and imaged in the presence of the membrane impermeable dye DRAQ7 (pink). YFP-Parkin is shown in grey. Frames were collected every 15 mins and saved for playback at 3 frames/sec.

**Movie 7.** **USP30 depletion sensitizes U2-OS cells to cell death induced by the BH3-mimetic ABT-737.** U2-OS cells were transfected for 72 hrs with either non-targeting siRNA (NT1, top and bottom left panels, or siRNA targeting USP30 (D1 and D3). Cells were then treated with ABT-737 (10µM) and imaged in the presence of Alexa Fluor 350 conjugated Annexin V (blue) and DRAQ7 (pink). Frames were collected at 15min intervals and saved for playback at 3 frames/sec.

**Movie 8.** **USP30 depletion sensitizes MCF7 cells to cell death induced by the BH3-mimetic ABT-737.** MCF7 cells were transfected for 72 hrs with either non-targeting siRNA (NT1, top and bottom left panels, or siRNA targeting USP30 (D1 and D3). Cells were then treated with ABT-737 (10µM) either on its own or in combination with Epoxomicin (100nM, bottom left panel). Cells were imaged in the presence of Alexa Fluor 350 conjugated Annexin V (blue) and DRAQ7 (pink). Frames were collected at 15min intervals and saved for playback at 3 frames/sec.
