# Supplementary material for: USP30 deubiquitylates mitochondrial Parkin substrates and restricts apoptotic cell death
Source: EMBO Rep. 2015 Mar 4;16(5):618–27. doi: 10.15252/embr.201439820 (PMC4428036; doi:10.15252/embr.201439820)
Supplement: Supplementary file 16 — Review Process File [file embr0016-0618-sd16.pdf]

EMBO
reports

Manuscript EMBOR-2014-39820

USP30 deubiquitylates mitochondrial Parkin substrates and restricts apoptotic cell death

Jin-Rui Liang, Aitor Martinez, Jon D. Lane, Ugo Mayor, Michael J. Clague and Sylvie Urbe

Corresponding author: Sylvie Urbe and Michael J. Clague, University of Liverpool

Review timeline:

	Submission date:	31 October 2014
	Accepted:	11 February 2015

Transaction Report:

(Note: No Peer Review Process File is available with this article, as the authors have chosen not to make the review process public in this case.)

© European Molecular Biology Organization
